# Indium phosphide quantum dots: advanced synthesis, surface engineering, and biomedical applications in imaging, sensing, and therapy

**DOI:** 10.1039/d5ra06797a

**Published:** 2026-02-26

**Authors:** A. K. Kareem, Musallam Ahmed Salim Tabook, Esraa H. J. Mahdi, Ahmed Said Badawy, M. M. Rekha, Laxmidhar Maharana, P. Grace Kanmani Prince, Gaganjot Kaur, Hamza Fadhel Hamzah, Nadia Sarhan

**Affiliations:** a Biomedical Engineering Department, College of Engineering, Al-Mustaqbal University Hillah 51001 Babil Iraq; b Department of Mathematics and Sciences, College of Arts and Applied Sciences, Dhofar university Salalah-Sultanate of Oman Oman; c College of Pharmacy, The Islamic University Najaf Iraq; d Department of Computer Engineering, College of Computer Science, King Khalid University Al-Faraa Kingdom of Saudi Arabia; e Department of Chemistry and Biochemistry, School of Sciences, JAIN (Deemed to be University) Bangalore Karnataka India; f Department of Pharmaceutical Sciences, Siksha ‘O’ Anusandhan (Deemed to be University) Bhubaneswar Odisha-751030 India; g Department of Biomedical, Sathyabama Institute of Science and Technology Chennai Tamil Nadu India; h Department of Electronics and Communication Engineering, Chandigarh University Mohali Punjab India; i Department of Medical Laboratories Technology, AL-Nisour University College Baghdad Iraq; j Young Researchers and Elite Club, Tehran Branch, Islamic Azad University Tehran Iran msarhannadia@gmail.com

## Abstract

Indium phosphide quantum dots (InP QDs) are emerging as non-toxic, tunable, and biocompatible semiconductor nanomaterials with transformative potential in biomedical applications. This review highlights cutting-edge synthesis methods, including nonclassical nucleation and scalable production, alongside innovative surface engineering techniques such as ligand exchange, polymer coatings, and inorganic passivation to overcome challenges like surface defects and indium release. We explore their superior near-infrared (NIR) emission and low cytotoxicity, enabling high-sensitivity NIR bioimaging, resonance energy transfer-based biosensing, photodynamic therapy, drug delivery, and neural prosthetics. Compared to other nanoparticles, InP QDs offer enhanced NIR performance and regulatory compliance, making them economically viable for diagnostics and therapeutics. By addressing safety concerns through advanced shell designs and safer precursors, InP QDs pave the way for clinical translation. This review, with a focused emphasis on the biomedical translation of InP QDs, provides a structured roadmap for researchers and clinicians to harness their potential in next-generation healthcare solutions.

## Abstract 2

This review summarizes advances in InP quantum dot synthesis and surface engineering, highlighting strategies that enable low-toxicity NIR imaging, biosensing, and phototherapeutic applications for biomedical translation.

## Graphical abstract

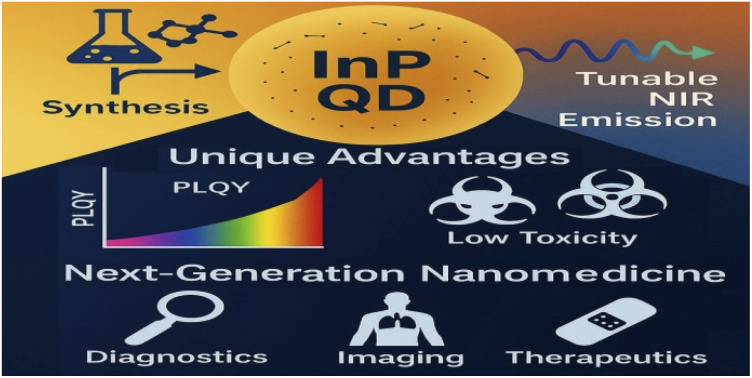

## 1 Introduction

Indium phosphide quantum dots (InP QDs) have emerged as a transformative class of semiconductor nanomaterials, distinguished by their non-toxic composition, tunable optical properties, and exceptional biocompatibility, positioning them as a compelling alternative to traditional heavy-metal-based QDs such as cadmium selenide (CdSe) and lead sulfide (PbS).^1–5^ These attributes have propelled InP QDs to the forefront of nanotechnology, particularly in biomedical applications, where their ability to emit in the near-infrared (NIR) spectrum (650–900 nm) and achieve high photoluminescence quantum yields (PLQYs) up to 97.7% enables advanced imaging, sensing, and therapeutic modalities.^6,7^ Unlike their II–VI counterparts, InP QDs offer reduced environmental and biological toxicity, aligning with the stringent safety requirements for clinical translation in precision nanomedicine.^8,9^

The synthesis of InP QDs has evolved significantly, with methods like hot-injection and one-pot synthesis providing precise control over particle size, morphology, and optical properties. Hot-injection techniques, involving rapid injection of phosphorus precursors such as tris(dimethylamino)phosphine ((DMA)_3_P) or tris(trimethylsilyl)phosphine (P(TMS)_3_) into indium-containing solvents, enable tailored nucleation and growth, achieving PLQYs as high as 97.7% for red emission at 680 nm through advanced surface passivation strategies.^10–12^ However, the use of toxic reagents like hydrofluoric acid (HF) for defect removal has prompted the development of safer alternatives, such as carboxylic-free synthesis using zinc chloride (ZnCl_2_), yielding InP/ZnSe/ZnSeS/ZnS structures with PLQYs of 96% and narrow full-width at half-maximum (FWHM) values of 41 nm. One-pot methods, while scalable, often compromise on PLQY due to less controlled nucleation kinetics, highlighting the need for innovative precursors like non-pyrophoric acylphosphines to enhance uniformity and safety.^13,14^ Doping strategies, including aluminum or neodymium incorporation, further refine optical performance by reducing lattice strain and enabling emission tuning from 470 to 627 nm, critical for applications in bioimaging and optoelectronics.^7,10^

Surface engineering is pivotal to the functionality of InP QDs, addressing challenges such as oxidation, aggregation, and non-radiative recombination in aqueous and biological environments. Ligand exchange with hydrophilic molecules like oleylamine (OAm) or sulfide ions enhances water dispersibility and PLQY by up to 50-fold, while polymer coatings, such as polyethylene glycol (PEG), ensure colloidal stability and biocompatibility for *in vivo* applications. Inorganic passivation with silica or metal oxides provides robust protection against environmental degradation, though it may impact quantum confinement.^15,16^ Advanced strategies, including amine-halide co-passivation and biotemplating with aptamers, optimize surface chemistry for specific biomedical needs, achieving three-month stability in aqueous media and enabling high-specificity bacterial detection. These modifications mitigate surface defects, such as dangling bonds and oxidative phosphorus species, which are critical barriers to achieving high PLQY and long-term stability.^4,15^

The biomedical applications of InP QDs are vast, leveraging their NIR emission and high photostability for multiplexed bioimaging, resonance energy transfer (RET)-based biosensing, photodynamic therapy (PDT), and drug delivery. In bioimaging, InP/ZnSe/ZnS QDs with PLQYs of 57% enable deep-tissue visualization of lymph nodes, critical for cancer staging, while antibody or aptamer conjugation enhances targeting specificity for pancreatic cancer cells and bacterial membrane proteins. In biosensing, cationic InP/ZnS QDs achieve 60% RET efficiency, detecting biomolecular interactions with a quenching constant of ∼10^5^ M^−1^ s^−1^, and aptamer-functionalized QDAPTs offer detection limits of ∼10^3^ CFU mL^−1^ for bacterial contaminants.^17,18^ For PDT, InP/ZnS QDs generate reactive oxygen species (ROS), achieving >99.9% bacterial inactivation, with applications in combating multidrug-resistant infections and cancer. Drug delivery systems utilizing carboxylated or PEGylated InP/ZnS QDs conjugated with cell-penetrating peptides demonstrate efficient cargo delivery with >90% cell viability, while type-II InP/ZnO QDs enable low-power neural photostimulation for retinal prostheses. These applications underscore the versatility of InP QDs in addressing unmet clinical needs.^19,20^

Despite these advancements, challenges persist in synthesis, stability, and clinical translation. Nonclassical nucleation and rapid precursor reactivity lead to heterogeneous size distributions, complicating monodispersity. Surface defects and indium release from degradation, particularly in carboxylated QDs, pose toxicity risks, with *in vivo* studies indicating liver and spleen accumulation for up to 90 days.^8,12^ Biomedical applications face hurdles in optimizing targeting specificity, ROS yield in hypoxic environments, and clearance kinetics. Strategies to overcome these limitations include safer precursors and advanced surface engineering with zwitterionic or pH-responsive ligands. Emerging applications, such as theranostic platforms, neural interfaces, point-of-care diagnostics, and immunotherapy monitoring, highlight the transformative potential of InP QDs, with NIR-II emission and stimuli-responsive conjugates poised to enhance precision medicine.^15,19^

Some review articles have previously addressed indium phosphide-based quantum dots from different perspectives, including synthetic optimization, optoelectronic performance, and device-oriented applications such as light-emitting diodes and photovoltaics. More recent reports have also begun to explore emerging biological and environmental applications of InP QDs, reflecting their growing relevance beyond traditional optoelectronic fields. However, in many of these studies, biomedical considerations are treated as ancillary extensions rather than as a central design framework.

In particular, key aspects required for biomedical translation—such as surface-chemistry-driven biocompatibility, long-term colloidal and chemical stability in physiological environments, *in vivo* biodistribution, clearance behavior, and toxicity-performance trade-offs—are often discussed in a fragmented or application-specific manner. A systematic integration of synthesis strategies, surface engineering, and biological performance remains limited. In this context, the present review aims to bridge this gap by focusing on how surface chemistry and structural design govern the biological behavior and translational potential of InP QDs in imaging, sensing, and therapeutic applications.^8–13^

**Fig. 1:**
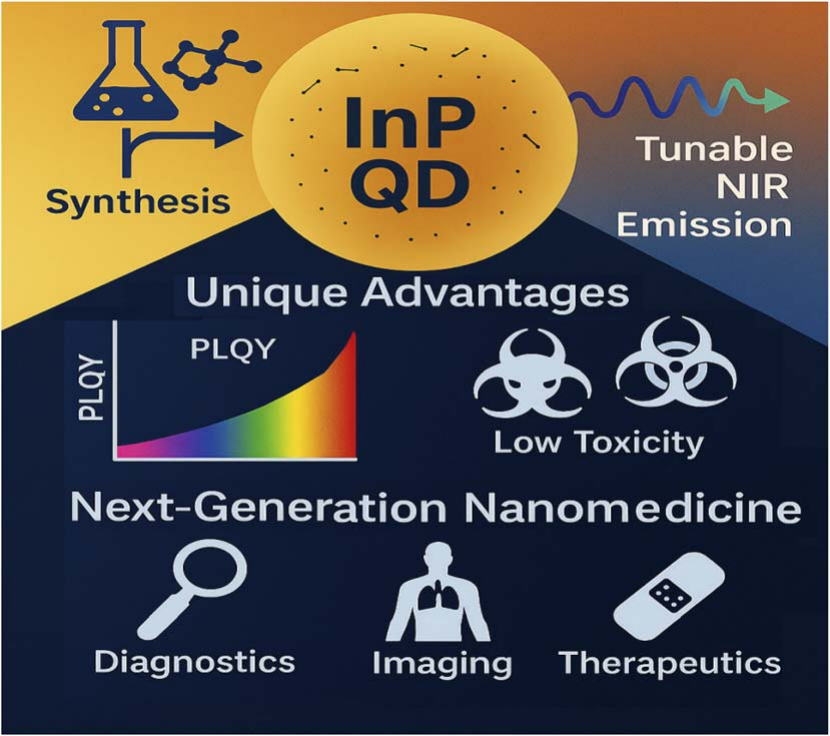
Graphical summary of InP quantum dots for biomedical applications, highlighting synthesis optimization, surface engineering, and biological performance for enhanced clinical translation.

This review represents an integrative review evaluation of InP QDs in biomedical applications, systematically synthesizing recent advancements in synthesis optimization, surface engineering, and biological performance. By critically analyzing strategies to enhance synthesis precision, improve surface stability, and ensure biocompatibility, it addresses the key challenges hindering clinical translation. The work highlights InP QDs' unique advantages, including tunable NIR emission, high PLQY, and low toxicity, positioning them as a safer and more versatile alternative to conventional heavy-metal-based QDs for next-generation diagnostics, imaging, and therapeutics in precision nanomedicine (Fig. 1). As a pioneering effort, this review not only consolidates current knowledge but also provides a critical foundation for future research to overcome existing limitations and unlock the full clinical potential of InP QDs.

## 2 Synthesis and optical properties of indium phosphide quantum dots

InP QDs have emerged as a promising class of semiconductor nanomaterials, offering a non-toxic alternative to cadmium- and lead-based QDs due to their tunable optical properties and environmental compatibility. Their small bandgap and high covalency enable fluorescence across the visible and near-infrared (NIR) spectrum, positioning them as candidates for applications in bioimaging, photocatalysis, optoelectronics, and light-emitting diodes (LEDs). Despite their potential, achieving high PLQY, narrow emission linewidths, and stability, particularly in aqueous environments, remains challenging compared to II–VI materials like CdSe.^4,11,15^

### 2.1 Synthesis methods

Although a wide range of synthetic strategies—such as microwave-assisted, stepwise thermal, solid-state, and electrochemical routes—have been explored for InP QD fabrication, colloidal synthesis methods remain dominant due to their superior control over particle size, morphology, and optical properties. Among these, hot-injection approaches are the most extensively studied, as they enable precise regulation of nucleation and growth kinetics through the rapid introduction of phosphorus precursors (*e.g.*, (DMA)_3_P or P(TMS)_3_) into a hot coordinating solvent containing indium sources. Such control has enabled the realization of high photoluminescence quantum yields (PLQYs) and relatively narrow emission linewidths in well-passivated InP-based core–shell structures.

For example, a hot-injection synthesis of InP/ZnS QDs employing HF-assisted interfacial oxide removal achieved a reported PLQY of 97.7% for red emission at 680 nm, highlighting the effectiveness of aggressive surface defect mitigation strategies. However, the toxicity and safety concerns associated with HF have motivated the development of alternative surface-engineering routes. Carboxylic-free synthesis protocols based on (DMA)_3_P and zinc chloride (ZnCl_2_) have enabled the preparation of multi-shell InP/ZnSe/ZnSeS/ZnS QDs exhibiting PLQYs up to 96% with emission linewidths around 41 nm. Similarly, mid-synthetic incorporation of zinc halides has been shown to suppress Ostwald ripening and passivate surface defects, resulting in PLQY values exceeding 70% and narrowed full-width at half-maximum (FWHM) values of approximately 40 nm.^21–24^

In parallel, one-pot synthesis strategies have gained attention due to their procedural simplicity and improved scalability, as they eliminate the need for rapid precursor injection. Variations in indium precursor concentration within single-step syntheses have produced InP and InP@ZnS QDs with tunable sizes and band-gap energies spanning 2.0–2.9 eV. While these methods are more amenable to large-scale production, they typically offer reduced kinetic control over nucleation, which can lead to lower PLQY or broader emission profiles compared to optimized hot-injection routes. Recent advances employing non-pyrophoric solid-state acylphosphine precursors have partially addressed these limitations by enabling more uniform particle formation while improving operational safety.

Doping-assisted approaches further expand the synthetic design space of InP QDs. Incorporation of Al^3+^*via* aluminum isopropoxide in one-pot reactions has yielded Al-doped InP/(Al)ZnS QDs with emission wavelengths ranging from 480 to 627 nm and PLQYs approaching 96%, attributed to reduced lattice strain and enhanced shell thickness. Conversely, Nd^3+^ doping during the nucleation stage has been shown to inhibit core growth, enabling blue-emitting InP/ZnS QDs at ∼470 nm, albeit with more moderate PLQY values.^25–29^ These examples collectively illustrate how dopant selection and incorporation timing can modulate both optical performance and emission color within simplified synthetic frameworks.

It is important to note that numerical values for PLQY, emission linewidth (FWHM), and related optical metrics reported across different synthesis studies are inherently sensitive to experimental and measurement conditions, including solvent environment, excitation wavelength, absorbance at the excitation wavelength, calibration standards, and data analysis protocols. As a result, the optical data summarized in Table 1 should not be interpreted as strictly equivalent benchmarks, but rather as representative indicators of performance trends associated with specific synthesis strategies. Despite this variability, consistent qualitative correlations emerge: approaches that enhance surface passivation, reduce lattice mismatch, or suppress uncontrolled precursor reactivity—such as multi-shell architectures, halide-assisted treatments, and controlled doping—generally yield higher PLQYs and narrower emission profiles. These observations underscore the importance of critically contextualizing reported optical metrics when comparing synthesis routes and highlight the need for standardized characterization protocols to improve reproducibility and cross-study comparability.

**Table 1:** Comparative summary of InP quantum dot synthesis strategies, optical performance, and key limitations

Synthesis method	Key precursors	Core/shell structure	Reported optical performance	Measurement sensitivity & limitations	Application relevance
Hot-injection	(DMA)_3_P or P(TMS)_3_ + in precursors	InP/ZnS or InP/ZnSe	High PLQY; relatively narrow FWHM	PLQY strongly dependent on solvent, excitation wavelength, and oxide removal efficiency	High-performance optoelectronics
Hot-injection + HF treatment	(DMA)_3_P + HF	InP/ZnS	PLQY up to 97.7%; emission ∼680 nm	HF enhances surface quality but raises safety and reproducibility concerns	Red-emitting QLEDs
Carboxylic-free synthesis	(DMA)_3_P + ZnCl_2_	InP/ZnSe/ZnSeS/ZnS	PLQY ∼96%; FWHM ∼41 nm	Optical quality sensitive to halide concentration and shell growth sequence	Efficient light-emitting devices
Mid-synthetic halide treatment	Zn halides	Multi-shell, Zn-rich	PLQY >70%; FWHM ∼40 nm	Linewidth varies with timing of halide addition and reaction temperature	Stability-enhanced QDs
One-pot synthesis	Indium myristate-based	InP or InP@ZnS	Moderate PLQY; size-dependent band gaps (2.0–2.9 eV)	Broader emission due to limited nucleation control; PLQY protocol-dependent	Scalable production
Solid-state precursor route	Acylphosphines	Core-only or thin shell	Excitonic absorption 460–600 nm	Optical metrics vary with precursor purity and heating profile	Safer laboratory synthesis
Al-doped one-pot	AIP dopant	InP/(Al)ZnS	PLQY up to ∼96%; tunable emission	PLQY influenced by dopant concentration and shell thickness	Full-color QLEDs
Nd^3+^-doped nucleation	Nd^3+^ dopant	InP/ZnS	Blue emission (∼470 nm); PLQY ∼44%	Reduced PLQY due to growth inhibition and defect introduction	Short-wavelength emitters
Microwave-assisted	Various In/P sources	InP/ZnS	Moderate PLQY	Optical variability linked to rapid heating and non-uniform growth	Scalable synthesis
Stepwise thermal	Thermally reactive precursors	InP@ZnS	Broader FWHM; moderate PLQY	Strong dependence on temperature ramping profile	Custom reaction control
Electrochemical	Electrochemical InP	Thin shell or none	Low PLQY	Limited size and surface control; measurement protocol sensitive	Exploratory applications

### 2.2 Nucleation and growth mechanisms

The nucleation and growth of InP QDs exhibit nonclassical behavior, meaning that their formation does not strictly follow the conventional nucleation-growth models commonly applied to II–VI semiconductor materials such as CdSe. This fundamental difference complicates the achievement of highly monodisperse particle size distributions. During high-temperature synthesis, the reaction pathway often involves kinetically persistent “magic-size” nanoclusters, which are unusually stable intermediate species with discrete atomic sizes. These nanoclusters temporarily arrest further growth and strongly influence surface chemistry and ligand coordination, ultimately affecting the size uniformity of the final QDs.^31^

A key challenge in InP QD synthesis arises from the rapid conversion of phosphorus precursors, which is driven by the small bandgap and high covalent character of InP. This fast reaction kinetics promotes heterogeneous nucleation events, leading to broader size distributions compared to II–VI QDs.^32^ To moderate this reactivity, zinc–phosphorus complexes, such as Zn–P intermediates formed in the presence of tris(trimethylsilyl)phosphine (P(TMS)_3_), have been introduced. These intermediates effectively reduce the reactivity of phosphorus species, slowing down nucleation and growth processes and thereby improving monodispersity.^33^ By buffering precursor reactivity, such complexes enable more controlled nucleation and the formation of QDs with narrower size distributions.

Trace impurities also play a critical role in determining growth kinetics and optical properties. For example, residual water in indium myristate precursors significantly suppresses size tunability, restricting the first excitonic absorption peak to approximately 550 nm, compared to about 620 nm under rigorously water-free conditions. This effect is attributed to hydroxylation reactions that interfere with surface growth at later stages.^34^ Similarly, free hydroxide ions alter surface chemistry and further inhibit controlled crystal growth.

Post-synthesis processing strategies are therefore essential for improving product quality. Size-selective agglomeration (SSA) is a widely used purification method that exploits differences in colloidal stability to separate InP/ZnS core/shell QDs from ZnS byproducts formed during shell growth. Using ethanol as a poor solvent, SSA enables the fractionation of QDs into multiple size classes.^35^ Notably, smaller InP/ZnS QDs isolated by this method exhibit higher photoluminescence quantum yields (PLQYs), reflecting a reduced density of surface and structural defects. In parallel, machine learning-based approaches have recently been applied to correlate synthetic parameters with optical properties, achieving mean absolute errors as low as 11.46 nm in predicting emission wavelengths.^36^ Such data-driven models provide valuable guidance for optimizing nucleation and growth conditions. Overall, these studies highlight that precise control over precursor chemistry, rigorous impurity management, and effective post-synthesis purification are all critical for regulating nucleation and growth processes and for producing high-quality, monodisperse InP quantum dots.

Table 2 synthesizes current understanding of the nucleation and growth mechanisms governing InP quantum dot formation and highlights why achieving monodispersity remains more challenging than for II–VI systems. A defining feature is the nonclassical nucleation pathway involving kinetically persistent magic-size nanoclusters, which act as stable intermediates and strongly influence final particle size distribution and surface chemistry. The extreme reactivity of commonly used phosphorus precursors further exacerbates heterogeneous nucleation, leading to broad size distributions unless precursor chemistry is carefully moderated. The formation of Zn–P complexes represents an effective strategy to attenuate precursor reactivity, thereby slowing growth kinetics and improving monodispersity. Table 2 also emphasizes the critical role of trace impurities, particularly water and hydroxide ions, which can severely limit size tunability and disrupt late-stage growth. Post-synthesis purification methods, such as size-selective agglomeration, emerge as essential tools for separating high-quality InP/ZnS QDs from shell byproducts and improving optical performance. Finally, data-driven approaches, including machine learning models, illustrate a growing shift toward predictive synthesis, offering quantitative guidance for optimizing nucleation and growth. Together, the trends summarized in Table 2 highlight that precise control over precursor reactivity, impurity levels, and post-synthetic processing is indispensable for reproducible, high-quality InP quantum dot synthesis.

**Table 2:** Nucleation and growth mechanisms of InP QDs

Aspect	Details	Key findings	Challenges	Ref.
Nucleation behavior	Nonclassical nucleation with magic-size nanoclusters as stable intermediates	Influences surface chemistry and ligand interactions, affecting QD uniformity	Heterogeneous nucleation due to rapid precursor conversion	31
Precursor reactivity	High reactivity of phosphorus precursors (*e.g.*, P(TMS)_3_)	Zn–P complexes reduce reactivity, promoting controlled nucleation and monodispersity	Broad size distributions due to rapid precursor conversion	32 and 33
Impurity effects	Water and hydroxide ions in indium myristate precursors	Limits size tunability (absorption peak at ∼550 nm *vs.* 620 nm in water-free conditions)	Hydroxylation affects growth kinetics at later stages	34
Post-synthesis purification	SSA with ethanol	Separates InP/ZnS QDs from ZnS byproducts, improving PLQY in smaller fractions	Byproduct formation during shelling	35
Data-driven optimization	Machine learning models for synthetic parameter prediction	Predicts emission wavelength with mean absolute error of 11.46 nm, aiding nucleation control	Requires extensive datasets for accuracy	36

**Fig. 2:**
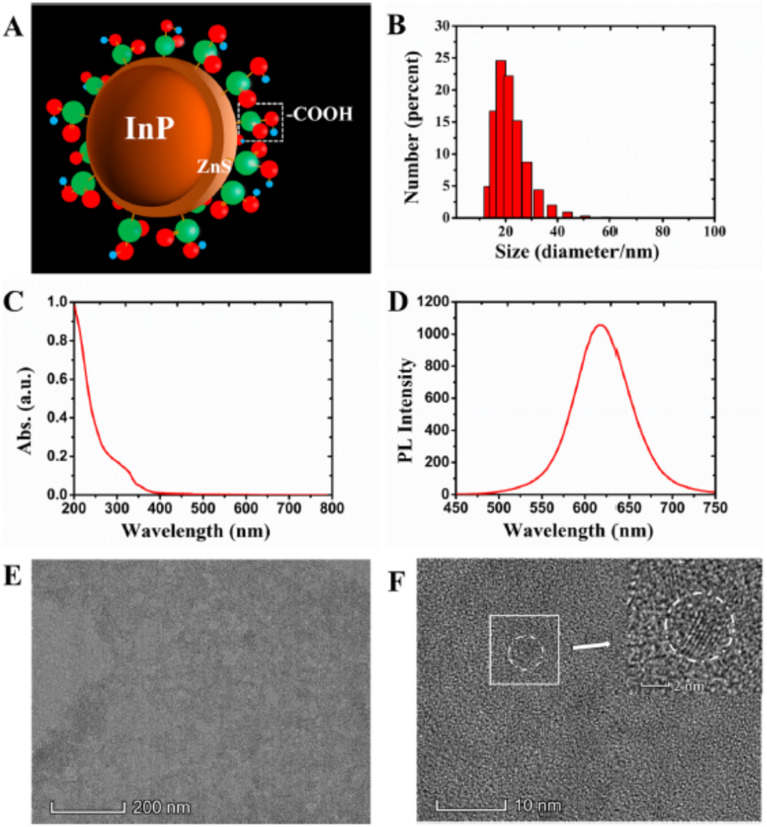
Structural and optical characterization of InP/ZnS quantum dots. (A) Schematic illustration of the InP/ZnS core–shell architecture with surface ligands; (B) hydrodynamic diameter distribution measured by dynamic light scattering (DLS); (C) UV-vis absorption spectrum; (D) photoluminescence (PL) emission spectrum; (E) low-magnification transmission electron microscopy (TEM) image showing particle dispersion (scale bar: 200 nm); (F) high-resolution TEM image resolving individual quantum dots (scale bar: 10 nm), with inset showing a magnified view of selected particles (scale bar: 2 nm). Adapted with permission from Ye *et al.*, *ACS Applied Bio Materials*, 2019, **2**, 4193–4201. © 2019 American Chemical Society.

Fig. 2 presents the fundamental structural, size, and optical characterizations of representative InP/ZnS quantum dots. Panel A schematically illustrates the core–shell configuration, consisting of an InP core encapsulated by a ZnS shell and capped with surface ligands. The hydrodynamic diameter distribution obtained by dynamic light scattering (Panel B) reveals the overall particle size and dispersion in solution, reflecting the size uniformity achieved through the applied synthesis and purification procedures. The UV-vis absorption spectrum (Panel C) and photoluminescence (PL) emission profile (Panel D) confirm the optical properties of the InP/ZnS QDs, including their characteristic absorption features and emission wavelength, which are key indicators of quantum confinement and surface passivation quality. Transmission electron microscopy (TEM) images in Panels E and F provide direct visualization of the morphology and nanoscale dimensions of the InP/ZnS quantum dots at different magnifications. The low-magnification image demonstrates good particle dispersion, while the high-resolution TEM image and inset clearly resolve individual QDs, confirming their nanometer-scale size. These characterization results collectively verify the physical dimensions, morphological uniformity, and optical performance of the synthesized InP/ZnS QDs, establishing a reliable experimental basis for evaluating synthesis outcomes without attributing the observed properties to specific nucleation or growth mechanisms.

### 2.3 Optical properties and tunability of emission wavelengths

InP QDs are valued for their tunable emission, spanning the visible to NIR spectrum, driven by quantum confinement effects. Emission wavelengths from 480 to 845 nm have been achieved by varying core size and shell thickness in InP/ZnSe/ZnS core/shell/shell structures, with consistent FWHM values of ∼0.32 eV.^37^ This tunability, particularly in the first optical tissue window (650–900 nm), enhances their applicability in deep-tissue bioimaging. High absorptivity across UV, visible, and NIR wavelengths supports efficient photoexcitation, making InP QDs versatile for photocatalytic and optoelectronic applications.^38^ For instance, InP/ZnS QDs degraded 60% of caffeic acid in water treatment under visible light, leveraging their strong absorption.^39^ Historically, InP QDs exhibit lower PLQY than CdSe-based QDs due to surface traps and lattice mismatch. Surface passivation with bifunctional metal oleates, replacing toxic HF etching, removes native oxides and narrows PL bandwidth by 20%, achieving a PLQY of up to 50%.^32^ Water-activated ligand exchange with sulfide ions enhances water dispersibility, improving photocatalytic performance.^39^ Doping strategies further optimize optical properties. Aluminum doping in ZnSeS shells enhances radiative recombination, yielding green InP/ZnSeS/ZnS QDs with a PLQY of 96% and an FWHM of 37 nm.^40^ Copper doping in ZnSe shells introduces impurity states, improving charge separation in photoelectrochemical cells with photocurrent density up to 8.7 mA cm^−2^.^41^ Nd^3+^ doping anchors at the InP surface, stabilizing small cores for blue emission at 470 nm.^27^ These advancements demonstrate significant progress in overcoming optical limitations through surface engineering and compositional tuning.

**Fig. 3:**
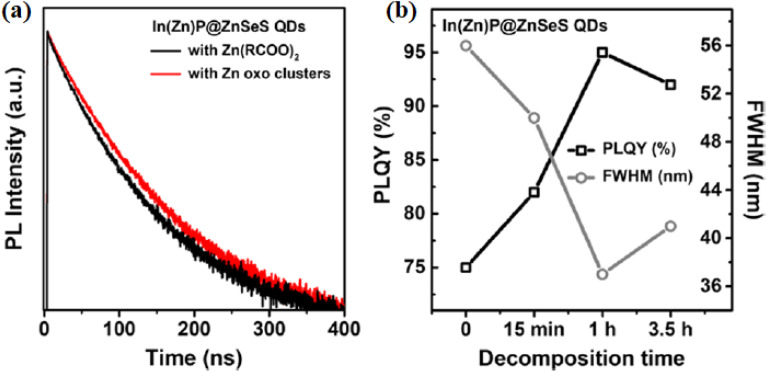
Optical characteristics of In(Zn)P@ZnSeS QDs: (a) time-resolved PL decay curves with Zn(RCOO)_2_ (black) and Zn oxo clusters (red), (b) PLQY (%) and FWHM (nm) as functions of decomposition time (0, 15 min, 1 h, 3.5 h). *Adapted with permission from Kim et al., Chem. Mater.* 2020, **32** (7), 2795–2802. © 2020 American Chemical Society. Ref. 5.

The PL properties of In(Zn)P@ZnSeS QDs are significantly improved through the application of zinc oxo clusters, as outlined in the provided figures and document. Fig. 3a showcases the time-resolved PL (TRPL) decay curves for In(Zn)P@ZnSeS QDs synthesized using Zn(RCOO)_2_ (black) and Zn oxo clusters (red), revealing a longer average lifetime (*τ*_avg_) with Zn oxo clusters, which points to fewer trap states and better optical performance.^5^Fig. 3b highlights the PLQY and FWHM as they vary with decomposition time (0, 15 min, 1 h, 3.5 h), demonstrating that Zn oxo clusters maintain a high PLQY (up to 95%) and a tight FWHM (around 37–44 nm) over time, outshining conventional precursors. These advancements are linked to better-controlled reactivity with the phosphorus source, enhanced Zn alloying, and the creation of an oxidized buffer layer, as supported by MALDI-TOF and XPS analyses in the study.

#### 2.3.1 Measurement of photoluminescence quantum yield and energy transfer efficiency

(PLQY values reported for InP-based quantum dots are predominantly determined using relative measurement approaches under controlled excitation conditions. Excitation wavelengths are typically selected near the first excitonic absorption peak of the quantum dots to ensure efficient and uniform excitation while minimizing reabsorption effects. For visible-emitting InP QDs, excitation wavelengths in the range of 400–450 nm are commonly employed, whereas near-infrared-emitting systems are excited at longer wavelengths, typically between 480 and 520 nm.^21,22^ All measurements are generally performed at room temperature, and emission spectra are corrected for instrumental response prior to analysis.

Well-established fluorescent standards are used to enable quantitative comparison of PLQY values across different studies. Rhodamine 6G in ethanol (PLQY = 0.95) and quinine sulfate in 0.1 M H_2_SO_4_ (PLQY = 0.54) are widely adopted as reference standards for visible-emitting InP QDs, while IR-26 in dichloroethane (PLQY = 0.05) is typically used for near-infrared-emitting samples.^37,52^ PLQY values are calculated using the standard comparative equation, taking into account the integrated emission intensity, absorbance at the excitation wavelength (maintained below 0.1), and the refractive index of the solvent. This methodology ensures consistency with previously reported high-performance InP/ZnSe/ZnS and InP/ZnS quantum dot systems.^22,37,40^

To ensure reliable and reproducible PLQY measurements, samples and reference standards are measured under identical optical geometries and excitation conditions. Dilute solutions are employed to minimize inner filter effects, and reported PLQY values typically represent averages obtained from multiple independent measurements. Such standardized experimental precautions are essential for meaningful comparison of optical efficiencies across different synthesis routes, shell architectures, and surface-engineering strategies reported in the literature.

Resonance energy transfer (RET) efficiency in InP quantum dot-based biosensing platforms is primarily evaluated using steady-state photoluminescence quenching experiments. Measurements are conducted under donor-selective excitation conditions, typically within the 400–450 nm range, to suppress direct excitation of the acceptor species.^55,56^ RET efficiency (*E*) is calculated using the relation *E* = 1 − *F*_DA_/*F*_D_, where *F*_D_ and *F*_DA_ correspond to the donor emission intensity in the absence and presence of the acceptor, respectively. In several studies, time-resolved photoluminescence measurements are additionally employed to confirm energy transfer by comparing donor lifetimes before and after acceptor introduction, providing complementary validation of the RET process.^56^ The use of clearly defined excitation conditions, reference standards, and calculation methods for PLQY and RET efficiency enables quantitative comparison across diverse InP QD systems and biosensing configurations, including aptamer-functionalized probes and electrostatically assembled donor–acceptor architectures.^52,55,56^ By explicitly summarizing these measurement protocols, the present review provides a reproducible framework for evaluating optical efficiency and energy transfer performance in emerging InP quantum dot-based biomedical and optoelectronic applications.

### 2.4 Impact of shells and core/shell structures on optical performance

Core/shell architectures are pivotal in enhancing the optical performance and stability of InP QDs. Zinc sulfide (ZnS) shells passivate surface defects and protect against oxidation, but lattice mismatch with InP cores introduces strain. Incorporating a ZnSe intermediate shell in InP/ZnSe/ZnS structures reduces this mismatch, increasing PLQY by up to 10-fold and enabling brightness-matched green and red emitters for multiplexed imaging.^38^ Double-shell InP/ZnS/ZnS structures further enhance luminescence, achieving emission peaks at 680 nm and a six-fold increase in photocurrent density (4 × 10^−6^ mA cm^−2^) in photoelectrochemical applications.^42^ Gradient-alloyed ZnSeS shells in InP/ZnSeS/ZnS QDs suppress Auger recombination, yielding a PLQY of 86% and external quantum efficiency (EQE) of 16.3% in green QLEDs.^43^

Shell thickness and composition significantly influence optical properties. Inverted ZnSe/InP/ZnS structures allow emission tuning from 515 to 845 nm by adjusting InP shell thickness, maintaining consistent FWHM.^37^ Layer-by-layer aluminum modification during ZnSeS and ZnS shell growth increases PLQY to 96% and narrows FWHM to 37 nm by alleviating charge mismatch and passivating defects.^40^ Doping shells with iron single-atom catalysts enhances photoelectric conversion, achieving a 5.6-fold photocurrent enhancement in biosensing applications.^44^

**Fig. 4:**
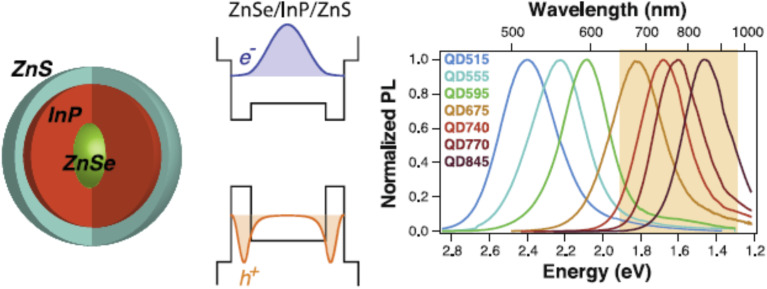
ZnSe/InP/ZnS core/shell/shell nanoparticles. ZnSe acts as a spacer for the InP shell, enabling longer, tunable emission wavelengths. Left to right: schematic, bandgap alignment with wave functions, and PL spectra with increasing InP deposition (3.4 nm ZnSe core, 0.75 nm InP shell; ZnS modeled as infinite well). Adapted with permission from Saeboe *et al.*, *Nano Lett.* 2021, **21** (7), 3271–3279. © 2021 American Chemical Society. Ref. 52.

The optical properties and structural design of InP/ZnSe/ZnS core/shell/shell nanoparticles are comprehensively depicted in Fig. 4, aligning with advanced strategies to optimize quantum dot performance. The schematic on the left illustrates the multilayer architecture, featuring a central InP core enveloped by a ZnSe spacer layer and an outer ZnS shell, which collectively mitigate lattice mismatch and enhance stability.^52^ The bandgap alignment diagram and corresponding normalized PL spectra for QDs with increasing InP deposition (QD515 to QD845) demonstrate tunable emission wavelengths exceeding traditional InP QD reports, ranging from 515 to 845 nm, with the ZnSe layer facilitating longer wavelengths and the ZnS capping modeled as an infinite well. These enhancements, driven by controlled shell thickness and composition, are consistent with improved PLQY and reduced Auger recombination, as evidenced by the electron and hole wave function distributions for a 3.4 nm ZnSe core with a 0.75 nm InP shell.

However, shelling can introduce byproducts, such as ZnS nanoparticles, which SSA effectively removes to improve homogeneity.^35^ Surface chemistry plays a critical role in stability. Biotemplating with aptamers ensures long-term stability (three months) in aqueous environments, enabling bacterial monitoring with high brightness.^45^ Encapsulation with CYTOP fluoropolymer renders InP/ZnSeS QLEDs waterproof, maintaining an EQE of 0.904% after 20 minutes of water immersion.^46^ These strategies highlight the synergistic role of shell design, doping, and surface passivation in optimizing InP QD performance for diverse applications. Table 3 presents optical properties and impact of core/shell structures of InP QDs.

**Table 3:** Optical properties and impact of core/shell structures of InP QDs

Aspect	Details	Performance metrics	Applications/challenges	References
Emission range	Tunable from 480 to 845 nm *via* core size and shell thickness	Consistent FWHM (∼0.32 eV) in InP/ZnSe/ZnS structures	Deep-tissue bioimaging; requires precise size control	37 and 38
Absorptivity	High across UV, visible, and NIR wavelengths	60% caffeic acid degradation in water treatment; supports efficient photoexcitation	Photocatalysis, optoelectronics; surface trap limitations	38 and 39
Surface passivation	Bifunctional metal oleates replace toxic HF etching	PLQY up to 50%, PL bandwidth narrowed by 20%	Bioimaging, LEDs; balancing passivation with toxicity concerns	32
Doping strategies	Al in ZnSeS shells; Cu in ZnSe shells; Nd^3+^ at InP surface	Al: PLQY 96%, FWHM 37 nm; Cu: 8.7 mA cm^−2^ photocurrent; Nd^3+^: Blue emission at 470 nm (PLQY 44%)	Biosensing, photoelectrochemical cells; dopant toxicity risks	40 and 41
Core/shell structures	ZnSe/ZnS shells reduce lattice mismatch	Up to 10-fold PLQY increase; brightness-matched green/red emitters	Complex synthesis; byproduct formation (*e.g.*, ZnS nanoparticles)	35 and 38
Double-shell/gradient shells	ZnS/ZnS or gradient-alloyed ZnSeS shells	ZnS/ZnS: 6-Fold photocurrent increase (4 × 10^−6^ mA cm^−2^); ZnSeS: PLQY 86%, EQE 16.3% in QLEDs	Precise composition control needed; Auger recombination	42 and 43
Surface stabilization	Aptamer biotemplating, CYTOP encapsulation	Three-month aqueous stability; EQE 0.904% after 20 min water immersion	Balancing stability with optical properties; cost of encapsulation	45 and 46

### 2.5 Comparative analysis and design guidelines for InP QD synthesis

Despite the wealth of reported synthetic strategies for InP quantum dots, a critical comparison reveals distinct trends and trade-offs that inform rational design. Hot-injection methods consistently achieve the highest photoluminescence quantum yields (PLQYs) and narrow emission linewidths (FWHM ∼37–44 nm), reflecting precise control over nucleation and growth kinetics. However, this performance advantage comes at the cost of operational complexity and potential hazards due to pyrophoric phosphorus precursors or aggressive treatments such as HF etching. In contrast, one-pot and solid-state precursor methods prioritize simplicity and scalability, yet generally yield broader emission profiles and lower PLQYs, indicating reduced kinetic control.^18,24^

Mid-synthetic halide treatments and multi-shell architectures partially mitigate these limitations, improving surface passivation and enhancing PLQY by up to 70%, while also narrowing FWHM. Doping strategies, including Al^3+^ and Nd^3+^ incorporation, further illustrate the potential to tune emission wavelength, reduce lattice strain, and compensate for intrinsic synthesis limitations, enabling high-performance optical properties even in simpler synthetic frameworks. Importantly, numerical PLQY comparisons across studies must be interpreted cautiously due to variations in measurement protocols, solvent environments, excitation wavelengths, and reference standards, emphasizing that qualitative trends are more reliable indicators than absolute values.^28,33^

Collectively, these observations highlight three emerging design principles for InP QD synthesis: (i) precise control of precursor reactivity, whether through hot-injection kinetics or complexation strategies, is essential for achieving high PLQY and narrow emission, (ii) surface engineering *via* shell growth, ligand modulation, or halide treatment is crucial to stabilize optically active sites and reduce defect-mediated non-radiative recombination, and (iii) synthesis route selection should be guided by application-specific requirements, balancing optical performance against safety, scalability, and reproducibility. Integrating these insights allows researchers to rationally choose or modify synthetic strategies, whether prioritizing high-performance optoelectronic devices or scalable, environmentally compatible production, ultimately bridging the gap between laboratory achievements and application-ready InP quantum dots.

## 3 Surface modification and stability

InP QDs have gained prominence as non-toxic alternatives to heavy-metal-based nanomaterials, offering tunable optical properties for applications in optoelectronics and biomedicine. However, their bare surfaces are prone to oxidation, aggregation, and defect formation, which compromise stability and performance. Surface modification strategies are critical to enhancing the colloidal, chemical, and photophysical stability of InP QDs, particularly in challenging aqueous and biological environments. These modifications also play a pivotal role in reducing surface defects and improving PLQY.^9,15^ This section examines the key surface modification approaches, their impact on stability in diverse media, and their contribution to defect passivation and PLQY enhancement, highlighting recent advancements and ongoing challenges.

### 3.1 Surface modifications

Surface modification of InP QDs involves the attachment of ligands, polymers, or inorganic layers to passivate surface atoms and tailor functionality. Common ligands include organic molecules such as oleylamine (OAm), oleic acid (OA), and thiols, which coordinate with surface indium or phosphorus atoms to prevent oxidation and aggregation. A water-activated ligand exchange process replaced oleylammonium chloride with OAm on aminophosphine-based InP QDs, enhancing PLQY by ∼50 times through coordination with Zn^2+^ ions, demonstrating the efficacy of ligand exchange in aqueous systems.^47^ Similarly, sulfide ion (S^2−^) exchange *via* a hot-injection method rendered InP QDs water-dispersible, enabling their use as visible-light photocatalysts for water treatment.^39^

Polymer coatings, such as PEG, provide steric stabilization and biocompatibility. PEGylated phospholipid micelles encapsulated InP/ZnSe/ZnS QDs, achieving water solubility and enabling NIR emission for multiplexed bioimaging in the 650–900 nm optical tissue window.^37^ Functional groups, such as carboxyl (–COOH), amine (–NH_2_), and hydroxyl (–OH), are introduced to enhance solubility and enable bioconjugation. InP/ZnS QDs with –COOH, –NH_2_, and –OH surface groups were synthesized and tested for biodistribution, showing rapid uptake in the liver and spleen without significant aggregation.^48^ Amine-halide co-passivation using OAm and chloride ligands stabilized InP QDs synthesized with diethylaminophosphine, allowing subsequent exchange with carboxylates or thiolates to tailor surface chemistry.^49^ These modifications highlight the versatility of surface engineering in addressing application-specific requirements.

**Fig. 5:**
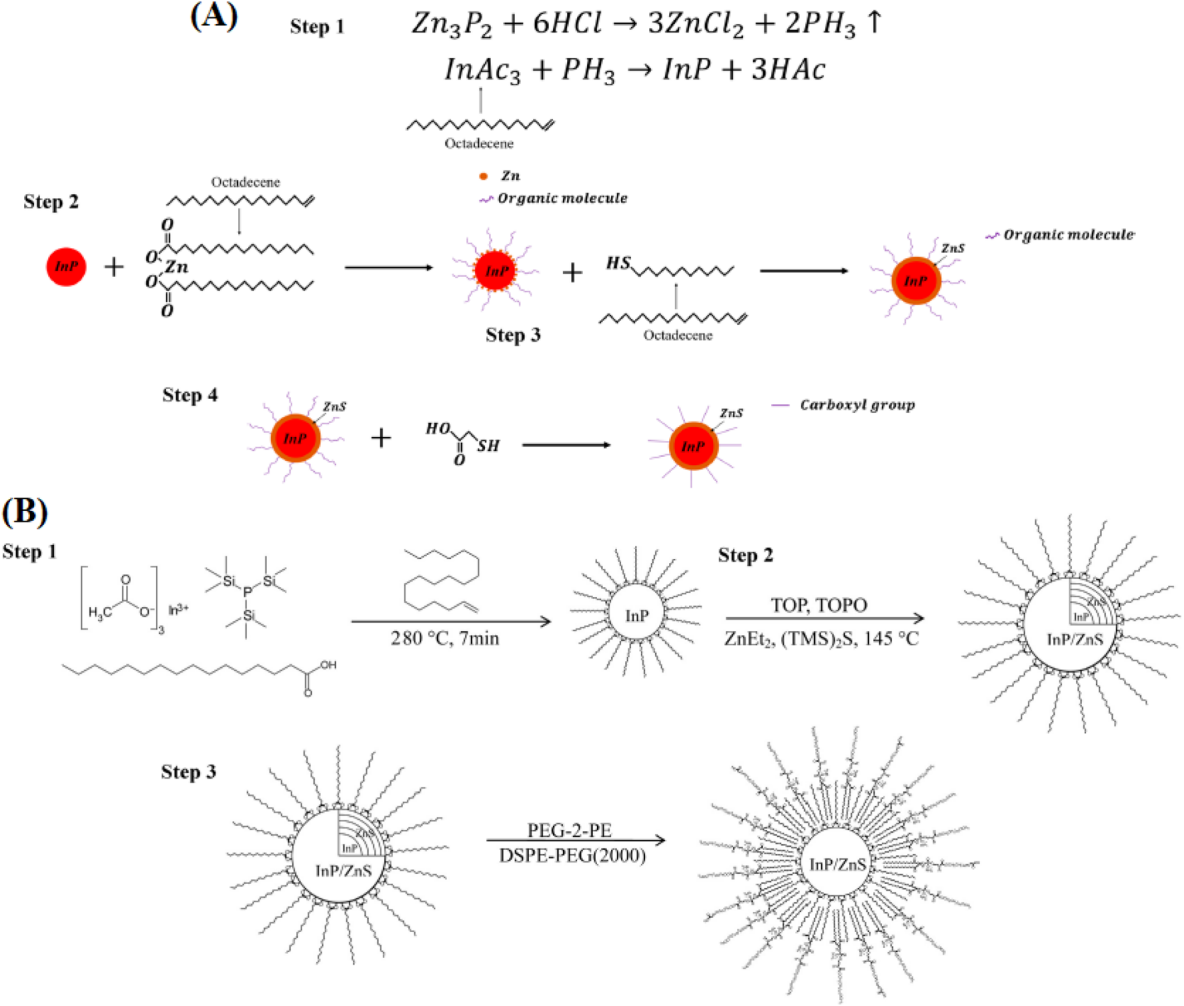
Synthesis and surface modification of InP and InP/ZnS QDs. (A) Steps: (1) Zn_3_P_2_ + 6HCl → 3ZnCl_2_ + 2PH_3_, InAc_3_ + PH_3_ → InP + 3HAc; (2) InP nucleation with Zn and organics; (3) HS- addition; (4) ZnS and carboxyl group. Reproduced with permission from Ye *et al.*, *ACS Appl. Bio Mater.* 2019, **2** (10), 4193–4201. © 2019 American Chemical Society. Ref. 68. (B) Steps: (1) thermal decomposition at 280 °C, 7 min; (2) ZnEt_2_ and (TMS)_2_S at 145 °C; (3) PEG-PE and DSPE-PEG(2000) coating. Adapted with permission from Liu *et al.*, *Colloids Surf. B: Biointerfaces* 2013, **111**, 162–170. © 2013 Elsevier. Ref. 61.

The synthesis and surface modification of InP and InP/ZnS QDs are detailed in Fig. 5, illustrating a multi-step process to enhance stability and functionality. In Panel A, Step 1 initiates with the reaction of Zn_3_P_2_ and HCl to form ZnCl_2_ and PH_3_, followed by InAc_3_ and PH_3_ reacting to produce InP and 3HAc, setting the foundation for QD formation. Step 2 depicts the nucleation of InP with Zn precursors and organic molecules in octadecene, followed by Step 3 where HS- ligands are introduced to form an InP core, and Step 4 shows the addition of ZnS and a carboxyl group to complete the shell structure. Panel B outlines a complementary synthesis route: Step 1 involves the thermal decomposition of indium myristate and sulfur precursors at 280 °C for 7 minutes, Step 2 introduces ZnEt_2_ and (TMS)_2_S at 145 °C to form InP/ZnS, and Step 3 employs PEG-PE and DSPE-PEG(2000) for polymer coating, enhancing water solubility and biocompatibility. These processes align with advanced ligand exchange and polymer encapsulation techniques, optimizing PLQY and enabling tailored surface chemistry for applications such as photocatalysis and bioimaging.

Inorganic passivation, such as silica or metal oxide coatings, offers robust protection against environmental degradation. Silica encapsulation of InP/ZnS QDs has been reported to enhance stability in aqueous media by forming a protective barrier against oxidation.^47^ Such strategies are particularly effective for long-term storage and harsh chemical environments, though they may increase particle size, potentially affecting optical properties.

### 3.2 Effect of surface modification on stability in aqueous and biological environments

Stability in aqueous and biological environments is critical for biomedical and environmental applications of InP QDs. Unmodified InP QDs are hydrophobic and susceptible to aggregation in polar solvents, limiting their utility. Ligand exchange with hydrophilic molecules addresses this challenge. Sulfide ion-exchanged InP QDs exhibited stable dispersion in water, degrading 60% of caffeic acid and 30% of 4-hydroxyphenylacetic acid under visible light irradiation, demonstrating photocatalytic stability.^39^ PEGylation significantly enhances colloidal stability by reducing non-specific interactions with biomolecules. PEGylated InP/ZnSe/ZnS QDs maintained stability for three months in aqueous solvents, enabling bacterial membrane protein detection with high selectivity.^50^ In biological environments, surface modifications mitigate protein corona formation and cellular uptake issues. InP/ZnS QDs with –COOH, –NH_2_, and –OH functional groups showed no significant aggregation in serum-rich media, with biodistribution studies indicating persistence in the liver and spleen for 28 days without abnormal behavior in mice. However, high-dose –COOH-modified QDs induced acute inflammation, highlighting the need for optimized surface chemistry.^48^ Biotemplating with aptamers further enhances biological stability. InP/ZnSe/ZnS-aptamer conjugates (QDAPTs) exhibited fast binding kinetics (<5 min) and maintained brightness in cellular extracts, enabling real-time imaging of bacterial membrane proteins.^45^

Environmental factors, such as pH and ionic strength, influence stability. InP/ZnS and CuInS/ZnS QDs coated with oleic acid, oleylamine, or octadecylamine showed increased phase transfer rates to aqueous media at lower pH and higher ionic strength, with amine-functionalized ligands being particularly sensitive to pH changes due to valence alterations.^51^ Encapsulation with CYTOP, an amorphous fluoropolymer, rendered InP/ZnSeS QLEDs waterproof, preserving an EQE of 0.904% after 20 minutes of water immersion, demonstrating exceptional stability for wearable electronics.^52^ These findings underscore the role of tailored surface modifications in ensuring robust performance in diverse environments. Table 4 outlines surface alterations and stability across aqueous and biological settings.

**Table 4:** Surface modifications and stability in aqueous and biological environments

Aspect	Details	Performance metrics	Applications/challenges	Ref.
Ligand exchange	OAm, sulfide ions (S^2−^) replace oleylammonium chloride	OAm: ∼50× PLQY increase; S^2−^: 60% caffeic acid degradation in water	Photocatalysis, biosensing; pH sensitivity of amine ligands	39 and 47
Polymer coatings	PEGylation with phospholipid micelles or lipoic acid	Three-month stability in aqueous media; NIR emission (650–900 nm) for bioimaging	Bioimaging; potential size increase affecting optical properties	50
Functional groups	–COOH, –NH_2_, –OH groups on InP/ZnS QDs	No aggregation in serum-rich media; liver/spleen uptake for 28 days	Biodistribution studies; high-dose –COOH induces inflammation	48
Inorganic passivation	Silica encapsulation	Enhanced stability against oxidation in aqueous media	Long-term storage; increased particle size impacts quantum confinement	47
Environmental stability	CYTOP fluoropolymer encapsulation; pH/ionic strength effects	EQE 0.904% after 20 min water immersion; stable dispersion at low pH/high ionic strength	Wearable electronics; amine ligands sensitive to pH changes	46 and 51
Biological stability	Aptamer biotemplating (QDAPTs); PEGylated QDs in serum-rich media	<5 min binding kinetics for bacterial proteins; no aggregation for 28 days	Bacterial detection; protein corona formation risks	48 and 50

### 3.3 Quantum yield

Surface defects in InP QDs, such as dangling bonds and oxidative phosphorus species, promote non-radiative recombination, reducing PLQY and limiting their performance in biomedical and optoelectronic applications. Ligand exchange with organic molecules like OAm or sulfide ions effectively passivates these defects. A water-activated ligand exchange process replaced oleylammonium chloride with OAm on aminophosphine-based InP QDs, increasing PLQY by approximately 50-fold through coordination with Zn^2+^ ions and red-shifting absorption by 15–55 nm, enabling fluorescence-based sensing in aqueous systems.^47^ Sulfide ion (S^2−^) exchange *via* a hot-injection method further mitigated oxidative defects, enhancing water dispersibility and photocatalytic efficiency under visible light.^39^ Metal ion coordination with Zn^2+^, Cd^2+^, or Al^3+^ stabilizes undercoordinated surface atoms, further reducing defects. Water-activated Zn^2+^ coordination boosted PLQY by 50 times, while Cd^2+^ achieved a 15-fold enhancement, both supporting ion detection applications by passivating dangling bonds.^47^ The incorporation of AIP during synthesis formed AlPO_*x*_ layers, minimizing surface oxidation and achieving a PLQY of 96% for orange emission, improving photostability for light-emitting diode (LED) applications.^26^ Amine–halide co-passivation using chloride ligands prevented thiolate-induced mid-gap states, preserving band-edge emission and yielding a PLQY of ∼50% without hazardous HF.^49^

**Fig. 6:**
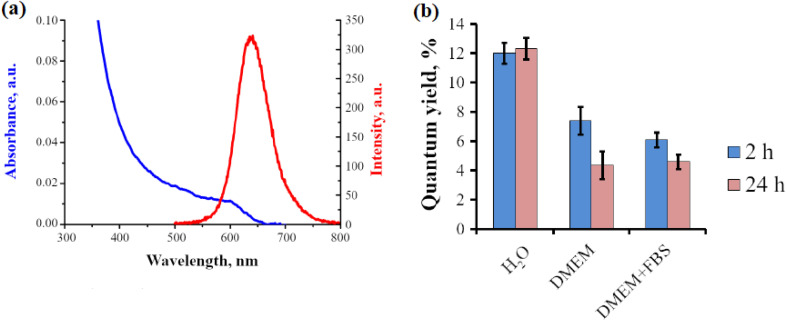
Photonic traits of InP QDs: (a) UV-Vis absorption (blue) and luminescence (red) profiles, (b) quantum efficiency (%) evaluation after 2 h (blue bars) and 24 h (red bars) across H_2_O, DMEM, DMEM + FBS, and DMEM media. Reproduced with permission from Litvinov *et al.*, *Int. J. Mol. Sci.* 2023, **24** (3), 2699. © 2023 MDPI. Ref. 9 Data-driven approaches optimize surface chemistry by predicting emission wavelengths with a mean absolute error of 11.46 nm, guiding ligand selection for defect passivation.^36^ However, challenges persist in ensuring ligand stability under physiological conditions, as amine-functionalized ligands are sensitive to pH changes due to valence alterations.^51^ Over-functionalization with bulky ligands may also disrupt quantum confinement, reducing PLQY. Developing pH-responsive or zwitterionic ligands could improve colloidal stability and support the application of InP QDs in demanding biomedical environments (Table 5).

The optical and stability characteristics of InP QDs are intricately detailed in Fig. 6, corroborating the critical role of surface defect passivation in enhancing their performance for biomedical and optoelectronic applications.^9^Fig. 6a presents the UV-Vis absorption (blue line) and PL spectra (red line), where the broad absorption profile peaking at 500–600 nm reflects quantum confinement in QDs sized 2–7 nm, while the sharp PL emission at ∼600 nm signifies efficient electron–hole recombination, tunable from 450–550 nm for smaller QDs to 650–700 nm for larger ones. This tunability, aligned with the solar spectrum, supports enhanced photocurrent generation and light harvesting, crucial for photoelectrochemical (PEC) processes like water splitting, and is consistent with the red-shift (15–55 nm) observed in water-activated ligand exchange with OAm. Fig. 6b compares QY under varying conditions, showing an initial QY of up to 12% in DMEM that declines after 24 hours (red bars), indicating surface degradation or ligand detachment, with moderate retention in DMEM + FBS. This aligns with the efficacy of sulfide ion (S^2−^) exchange and metal ion coordination (*e.g.*, Zn^2+^, Cd^2+^) in mitigating oxidative defects and stabilizing surface atoms, as well as the protective role of AlPO_*x*_ layers and amine-halide co-passivation in sustaining high PLQY (up to 96%) and photostability for LED applications.

**Table 5:** Reduction of surface defects and enhancement of quantum yield

Aspect	Details	Performance metrics	Challenges	Ref.
Ligand passivation	OAm, S^2−^ for dangling bond passivation	OAm: ∼50× PLQY increase, 15–55 nm red-shift; S^2−^: enhanced photocatalytic efficiency	Ligand stability under physiological conditions	39
Metal ion coordination	Zn^2+^, Cd^2+^, or Al^3+^ coordination with surface atoms	Zn^2+^: 50× PLQY increase; Cd^2+^: 15× PLQY increase; Al^3+^: PLQY 96% for orange emission	Potential toxicity of Cd^2+^; pH sensitivity of ligands	26 and 47
Amine–halide co-passivation	Chloride ligands with oleylamine	PLQY ∼50% without HF; prevents thiolate-induced mid-gap states	Over-functionalization may disrupt quantum confinement	49
Inorganic layers	AlPO_*x*_ layers *via* AIP	PLQY 96% for orange emission; minimizes surface oxidation	Complex synthesis process	26
Data-driven optimization	Machine learning for ligand selection	Predicts emission wavelength with 11.46 nm mean absolute error	Requires extensive datasets; ligand stability *in vivo*	36

Surface modification is a cornerstone of InP QD development, enabling stability in aqueous and biological environments, reducing surface defects, and enhancing PLQY. Ligand exchange with hydrophilic molecules, polymer coatings like PEG, and inorganic passivation with silica address colloidal and chemical stability, while metal ion coordination and doping mitigate non-radiative recombination. These strategies have achieved PLQYs up to 96% and robust performance in challenging media, paving the way for applications in biomedicine and optoelectronics. However, challenges remain in balancing functionalization with optical properties and ensuring long-term stability under physiological conditions. Continued advancements in surface engineering will be essential to realizing the full potential of InP QDs.

### 3.4 Critical comparison and emerging design principles

Although numerous studies report high PLQY, improved stability, and enhanced biocompatibility for InP-based QDs, a careful comparison across the literature reveals that these properties are strongly interdependent and sensitive to both surface chemistry and shell architecture. Optical performance and colloidal stability are rarely determined solely by core composition; rather, they emerge from a delicate balance among defect passivation, lattice mismatch mitigation, and ligand robustness. Therefore, reported improvements should not be interpreted as isolated achievements but as outcomes of carefully optimized trade-offs.^12,28^

Comparative analysis highlights that strategies achieving the highest PLQY, such as aggressive shell growth, gradient-alloyed shells, or heavy metal ion coordination, do not necessarily guarantee long-term chemical or colloidal stability. For instance, Al-doped or gradient-alloyed shells can achieve PLQY values exceeding 90%, yet these systems often require precise compositional control and may degrade under physiological conditions. Conversely, ligand-based surface passivation tends to provide superior aqueous and biological stability but results in more moderate PLQY enhancement. This intrinsic trade-off underscores the importance of tailoring surface engineering approaches to the intended application rather than focusing solely on maximizing PLQY.^30^

Apparent contradictions regarding toxicity and biocompatibility also emerge in the literature. While hydrophilic ligands or polymer coatings generally reduce toxicity, several studies report dose-dependent inflammatory responses, particularly for carboxyl-functionalized InP QDs. These findings suggest that surface charge density, ligand desorption, and long-term degradation products play more critical roles in biological interactions than previously assumed. Consequently, toxicity cannot be predicted solely based on the absence of heavy metals, highlighting a key unresolved challenge in InP QD design.

Shell engineering consistently appears as a dominant factor governing optical performance; however, discrepancies persist regarding optimal shell thickness and composition. ZnSe intermediate shells can effectively reduce lattice mismatch and enhance PLQY, but excessive shell growth may compromise size uniformity and reproducibility. Inverted or gradient shells offer tunable optical properties but increase synthetic complexity and batch-to-batch variability. Such contradictions reflect the lack of universal shell design rules, particularly for scalable systems intended for biomedical or optoelectronic applications.^44,49^

Taken together, comparative insights from the literature point to three emerging design principles for InP QDs:

(1) Balanced defect passivation is more critical than maximal shell thickness; overly aggressive shelling may improve PLQY temporarily but compromise stability.

(2) Surface ligand robustness under operational conditions is as important as initial optical performance; dynamic ligand desorption or exchange can reduce both PLQY and biocompatibility.

(3) Toxicity and stability must be evaluated dynamically; predictions based solely on composition or initial characterization are insufficient.

Future work should prioritize standardized testing conditions, long-term degradation studies, and integrative strategies that simultaneously optimize optical efficiency, colloidal and chemical stability, and biocompatibility. Addressing these challenges is essential for translating high-performance InP QDs from laboratory studies to reliable, real-world biomedical and optoelectronic applications.

### 3.5 Mechanistic classification of surface engineering effects in InP QDs

Surface engineering fundamentally governs the optical performance, chemical stability, and biomedical suitability of InP QDs. While improvements in PLQY and robustness are often attributed broadly to “defect passivation,” this term encompasses multiple mechanistically distinct processes. Recognizing these differences is essential for understanding experimental outcomes and for rationally designing InP QDs for specific applications. The effects of surface modification can be classified into four main mechanisms: chemical passivation, dielectric confinement, suppression of surface oxidation, and modulation of surface charge and ligand dynamics.

#### 3.5.1 Chemical passivation

Chemical passivation refers to the direct coordination of ligands or metal ions with undercoordinated surface atoms on InP QDs. Ligands such as oleylamine (OAm), thiols, and phosphine derivatives, as well as metal ions like Zn^2+^, Al^3+^, or Cd^2+^, bind to dangling bonds on indium or phosphorus sites, saturating trap states and eliminating mid-gap energy levels. This mechanism effectively suppresses non-radiative recombination pathways, restoring band-edge emission and improving PLQY. The extent of enhancement depends strongly on ligand density, binding strength, and the stability of the ligand-QD interface under environmental conditions.^31,37^

Experimentally, chemical passivation has been shown to yield dramatic increases in PLQY. For example, water-activated ligand exchange replacing oleylammonium chloride with OAm in aminophosphine-based InP QDs enhanced PLQY by approximately 50-fold while also red-shifting absorption by 15–55 nm. Similarly, sulfide ion (S^2−^) exchange reduced oxidative defects, improving water dispersibility and photocatalytic efficiency. Metal ion coordination further stabilizes undercoordinated surface atoms: Zn^2+^ coordination provided up to 50× PLQY enhancement, Cd^2+^ coordination yielded a 15× increase, and Al^3+^ incorporation achieved PLQY of 96% for orange emission.^42–44^ These improvements highlight the crucial role of chemical passivation in optimizing optical properties for both bioimaging and optoelectronic applications, while also emphasizing the need to balance ligand stability and toxicity considerations.

#### 3.5.2 Dielectric confinement

Dielectric confinement arises when polymeric coatings, inorganic shells, or wide-bandgap materials modify the local dielectric environment of the QD surface. Unlike chemical passivation, traps may still exist chemically, but their effect on exciton dynamics is mitigated by reduced coupling between charge carriers and surface states. This mechanism enhances radiative recombination efficiency and photostability without necessarily eliminating all surface defects.

Examples include PEGylated polymer coatings and silica shells, which increase exciton lifetimes and stabilize PL under prolonged illumination or in polar solvents. PEG-coated InP/ZnS QDs maintained colloidal stability in aqueous media for over three months while preserving near-infrared emission for bioimaging applications. Silica encapsulation, by increasing the effective dielectric barrier, protected QDs against photooxidation and hydrolysis, enabling long-term storage and operation in harsh chemical environments.^35–39^ These strategies are particularly valuable when high photostability is required under continuous excitation or in physiological media, highlighting the complementary nature of dielectric confinement to chemical passivation.

#### 3.5.3 Suppression of surface oxidation

Oxidation suppression targets long-term chemical and biological stability by preventing the formation of oxidized phosphorus species and indium leaching. Inorganic shells, oxide-forming interlayers, or multi-shell architectures serve as physical diffusion barriers that limit the access of oxygen and water to the QD core. While this mechanism may not maximize immediate PLQY, it is essential for maintaining colloidal integrity, photostability, and biocompatibility over extended periods.

For instance, AlPO_*x*_ layers formed *via* aluminum isopropoxide (AIP) incorporation during shell growth minimized surface oxidation, achieving a PLQY of 96% for orange emission while enhancing long-term photostability. Silica-coated InP/ZnS QDs retained structural integrity in aqueous environments over weeks, preventing aggregation and hydrolytic degradation. By stabilizing the surface chemically and physically, oxidation suppression ensures consistent optical performance and reduces potential cytotoxicity, enabling the use of InP QDs in biomedical imaging and environmental sensing applications.^42–46^

#### 3.5.4 Modulation of surface charge and ligand dynamics

Dynamic ligand exchange and surface charge modulation represent a complementary mechanism that influences colloidal stability, cellular interactions, and biological compatibility. Ligands with charged or zwitterionic functional groups can reduce non-specific protein adsorption, mitigate aggregation, and improve dispersion in physiological media. Controlling ligand density, exchange kinetics, and surface charge allows fine-tuning of QD interactions with solvents, biomolecules, and cellular membranes.

For example, aptamer-functionalized InP/ZnSe/ZnS QDs exhibited rapid binding (<5 min) to bacterial membrane proteins while maintaining colloidal stability in serum-rich media for over 28 days. Similarly, pH- or ion-responsive ligands stabilized QDs under varying biological conditions without compromising PLQY. This mechanism is particularly critical in biomedical applications, where ligand desorption or aggregation can reduce both optical performance and biocompatibility, and provides a framework for rational design of QDs for targeted imaging or therapeutic delivery.

## 4 Biomedical applications

The biomedical applications of InP QDs leverage their tunable emission, high photostability, and reduced heavy-metal toxicity; however, the performance requirements and translational challenges vary significantly across application domains. To avoid repetition of general photophysical principles discussed in earlier sections, this section focuses on application-specific design considerations, including signal-to-noise constraints in imaging and biosensing, targeting specificity at the cellular and tissue levels, and stability under *in vivo* and therapeutic conditions. Each subsection emphasizes how surface engineering, nanostructure design, and excitation strategies must be tailored to meet the distinct functional demands of diagnostics, sensing, therapy, and biointerfacing.

### 4.1 Bioimaging

Core/shell and core/shell/shell architectures play a central role in enabling InP quantum dots (QDs) for bioimaging by simultaneously improving optical efficiency, photostability, and spectral tunability. In particular, InP/ZnSe/ZnS QDs have demonstrated tunable emission spanning the visible to near-infrared (NIR) region (515–845 nm) with a photoluminescence quantum yield (PLQY) of up to 57% in aqueous media.^52^ Such spectral flexibility enables multiplexed imaging, as demonstrated in lymph node mapping in murine models, where multiple lymphatic drainage pathways were resolved simultaneously—an important capability for cancer staging and metastasis assessment. Inverted heterostructures such as ZnSe/InP/ZnS further extend emission into the NIR by alleviating the InP growth bottleneck, thereby enhancing tissue penetration and reducing autofluorescence interference.

Beyond nanocrystal architecture, surface functionalization is essential for achieving molecular specificity in targeted bioimaging. Antibody-conjugated InP/ZnS QDs targeting claudin-4 and prostate stem cell antigen (PSCA) have demonstrated selective uptake in pancreatic cancer cell lines *via* receptor-mediated endocytosis, with minimal non-specific binding and stable fluorescence over 24 h.^53^ These results highlight the potential of InP QDs for early cancer detection in tissues where high contrast and low background signals are critical. Similarly, functionalization with (3-carboxypropyl)triphenylphosphonium (TPP) has enabled long-term tracking of mitochondrial dynamics in cancer cells, providing insights into fission and fusion processes that are central to cellular metabolism and disease progression.^54^

**Fig. 7:**
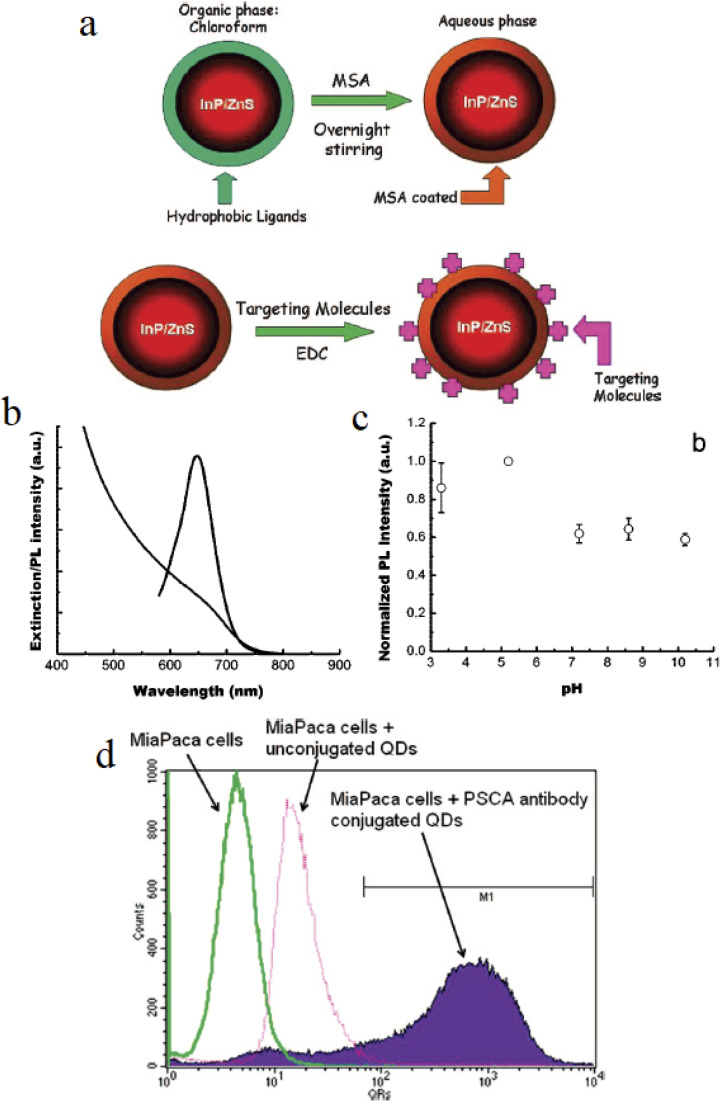
Imaging pancreatic cancer with InP/ZnS QDs: (a) phase transfer from organic (chloroform) to aqueous phase using MSA coating and targeting molecule conjugation with EDC, (b) extinction spectrum, (c) PL intensity stability across pH 3.3–10.8, (d) flow cytometry of MiaPaCa cells with unconjugated and PSCA antibody-conjugated QDs. Reproduced with permission from Yong *et al.*, *ACS Nano* 2009, **3** (3), 502–510. © 2009 American Chemical Society. Ref. 53.

Fig. 7 schematically illustrates the surface modification strategy and optical performance of targeted InP/ZnS QDs. Hydrophobic QDs are rendered water-dispersible through ligand exchange with mercaptosuccinic acid (MSA), followed by conjugation of targeting ligands using EDC chemistry, enabling receptor-specific cellular uptake.^53^ Optical characterization demonstrates broad absorption across the visible-NIR region and stable photoluminescence over a wide pH range, supporting their robustness under physiological conditions. Flow cytometry measurements further confirm a pronounced fluorescence shift for antibody-conjugated QDs compared to unconjugated controls, consistent with specific targeting and minimal background uptake.

In addition to cancer imaging, InP-based QDs have been applied to high-resolution bacterial imaging. PEGylated InP/ZnSe/ZnS aptamer-functionalized QDs exhibit rapid binding kinetics (<5 min) to bacterial membrane proteins and NIR emission suitable for imaging in scattering biological media.^55^ Their long-term aqueous stability (up to three months) and compatibility with handheld imaging devices highlight their potential for point-of-care diagnostics.

Despite these advances, several challenges remain for InP QD bioimaging. Achieving consistently high PLQY in the NIR-II window (1000–1700 nm), minimizing non-specific interactions in complex biological environments, and maintaining *in vivo* stability over extended imaging durations are ongoing hurdles. Furthermore, scalable synthesis with reproducible optical and surface properties is essential for clinical translation. Future strategies integrating InP QDs with plasmonic or upconversion nanostructures may further enhance signal-to-noise ratios in deep-tissue imaging applications.

### 4.2 Biosensing

InP quantum dots have emerged as versatile platforms for biosensing owing to their tunable emission, photostability, and ability to interface with diverse biomolecular recognition elements. In biosensing applications, performance is governed less by absolute optical brightness and more by signal-to-noise ratio, response kinetics, and robustness in complex biological matrices. Accordingly, InP QD-based biosensors have been developed using resonance energy transfer (RET), aptamer recognition, and hybrid nanocomposite architectures to achieve sensitive and selective detection.

Cationic InP/ZnS QDs prepared *via* ligand exchange with quaternary ammonium ligands have demonstrated efficient RET interactions with anionic dye acceptors under physiological conditions.^56^ These systems exhibit RET efficiencies of approximately 60% and bimolecular quenching constants on the order of 10^5^ M^−1^ s^−1^, enabling real-time monitoring of biomolecular interactions such as protein-ligand binding. The strong electrostatic association between donor and acceptor species enhances sensitivity, making these QDs suitable for high-throughput screening assays.

**Fig. 8:**
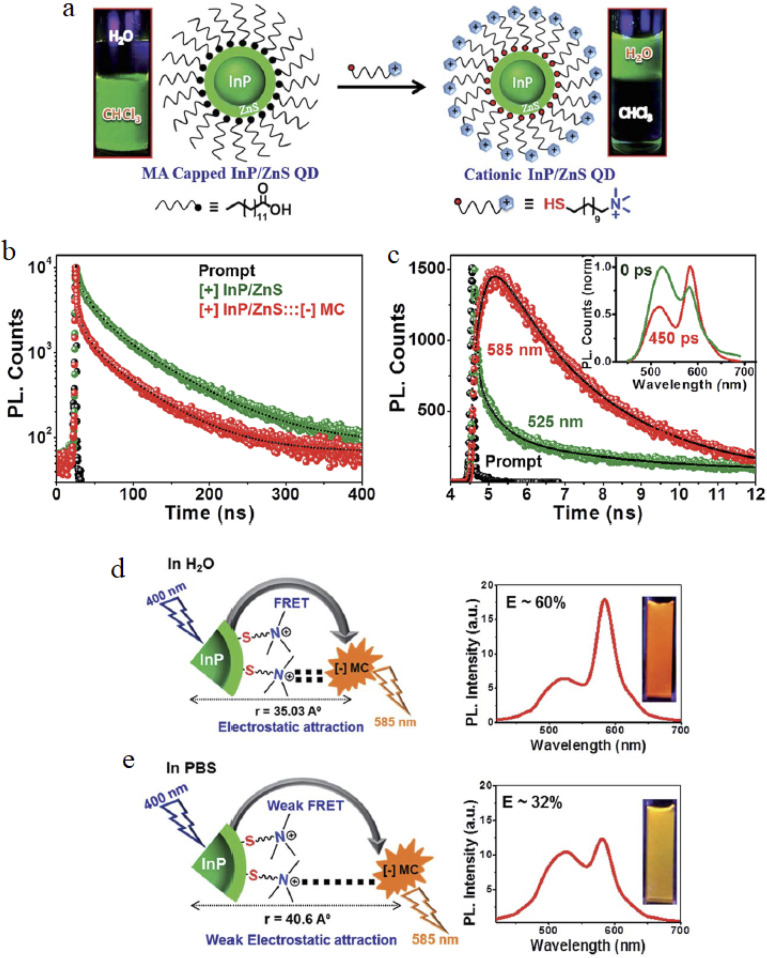
Synthesis and photoluminescence (PL) of cationic InP/ZnS QDs *via* TMA ligand exchange. (a) Transition from MA-capped to cationic QDs. (b) PL decay showing ∼80% intensity retention. (c) PL decay with MC dye, ∼60% RET at 585 nm. (d) RET in H_2_O, 60% efficiency, 35.03 Å distance. (e) RET in PBS, 32% efficiency, 40.6 Å distance due to salt screening. Reproduced with permission from Devatha *et al.*, *Chem. Sci.* 2017, **8** (5), 3879–3884. © 2017 The Royal Society of Chemistry. Ref. 56.

Fig. 8 summarizes the synthesis and photophysical behavior of cationic InP/ZnS QDs used in RET-based biosensing. Ligand exchange from myristic acid to trimethylammonium-functionalized ligands produces water-soluble, positively charged QDs while retaining approximately 80% of their original photoluminescence intensity. RET efficiency is strongly influenced by the ionic environment: high efficiencies are observed in water due to strong electrostatic interactions, whereas salt-induced charge screening in buffered solutions increases donor–acceptor separation and reduces energy transfer efficiency. These observations underscore the importance of optimizing sensing conditions to maintain high sensitivity in physiological media.

Hybrid and multimodal biosensing platforms further extend the capabilities of InP QDs. Integration with upconversion nanoparticles (UCNPs) and enzyme-responsive polypeptide linkers has enabled sensitive detection of matrix metalloproteinase-2 (MMP2) in cancer cells, with NIR excitation minimizing background interference and improving detection limits.^57^ Similarly, aptamer-functionalized InP/ZnSe/ZnS QDs have demonstrated rapid (<5 min) and selective detection of bacterial membrane proteins, achieving limits of detection as low as ∼10^3^ CFU mL^−1^ in handheld diagnostic formats.^55^

InP QD-based nanocomposites have also been applied to high-throughput ligand screening and drug discovery. For example, mesoporous silica-InP/ZnS QD platforms functionalized with Hsp90α have enabled efficient screening of natural product extracts, identifying bioactive compounds with high extraction yields.^58^ While these examples highlight the versatility of InP QDs in biosensing, key challenges remain, including improving detection limits for ultra-low analyte concentrations, mitigating matrix effects in complex fluids, and ensuring reproducible functionalization at scale. Future developments combining InP QDs with microfluidics, automated data processing, and machine learning-assisted signal analysis may further enhance sensitivity and throughput for next-generation biosensing applications (Table 6).

**Table 6:** Bioimaging and biosensing applications

Application	Details	Performance metrics	Challenges	Ref.
Multiplexed bioimaging	InP/ZnSe/ZnS QDs with ZnSe shell for lymph node imaging	PLQY 57% in aqueous media; emission 515–845 nm; FWHM ∼0.32 eV	Optimizing PLQY in NIR-II; improving targeting specificity	52
Targeted cancer imaging	InP/ZnS QDs with anti-claudin-4/anti-PSCA antibodies	Stable fluorescence for 24 h; specific uptake in pancreatic cancer cells	Minimizing non-specific binding in complex matrices	53
Mitochondrial tracking	InP/ZnS QDs with TPP functionalization	Long-term tracking of mitochondrial dynamics in cancer cells	Ensuring photochemical stability in cellular environments	54
Bacterial imaging	PEGylated InP/ZnSe/ZnS-aptamer QDAPTs	<5 min binding kinetics; NIR emission for bacterial membrane protein imaging	Stability in serum-rich media	55
RET-based biosensing	Cationic InP/ZnS QDs with quaternary ammonium ligands	60% RET efficiency; quenching constant ∼10^5^ M^−1^ s^−1^ for anionic biomolecules	Minimizing non-specific binding; enhancing specificity	56
MMP2 cancer biosensing	UCNP-p@InP-cRGD biosensor with MMP2-sensitive polypeptide	Fluorescence shifts for MMP2 detection; cRGD enhances cancer cell targeting	Sensitivity in complex biological matrices	57
Bacterial biosensing	InP/ZnSe/ZnS-aptamer QDAPTs	Detection limit ∼10^3^ CFU mL^−1^ with handheld imaging device	Improving signal-to-noise ratio in complex environments	55
Ligand screening	InP/ZnS QD-mesoporous silica with Hsp90α	Identified Alisol F (8.19 µg g^−1^) with 76.2% extraction efficiency	High-throughput processing limitations	58

### 4.3 Photodynamic and antimicrobial therapy

InP QDs have gained increasing attention as photoactive agents for photodynamic therapy (PDT) and antimicrobial applications, owing to their ability to generate reactive oxygen species (ROS) under visible or NIR illumination while avoiding the heavy-metal toxicity associated with cadmium- or lead-based nanomaterials. In therapeutic contexts, performance is governed by ROS quantum yield, activation wavelength, and photostability under clinically relevant light doses, rather than absolute emission brightness.

Photoactivated InP/ZnS QDs have demonstrated efficient superoxide generation under NIR illumination (650–800 nm), achieving greater than 99.9% (>3 log) reduction of drug-resistant bacterial abscesses in murine infection models without observable systemic toxicity.^59^ NIR activation enables deeper tissue penetration and reduces collateral photodamage, which is critical for treating localized infections. The high photostability of these QDs supports sustained ROS production over extended illumination periods, distinguishing them from conventional organic photosensitizers that suffer from rapid photobleaching.

**Fig. 9:**
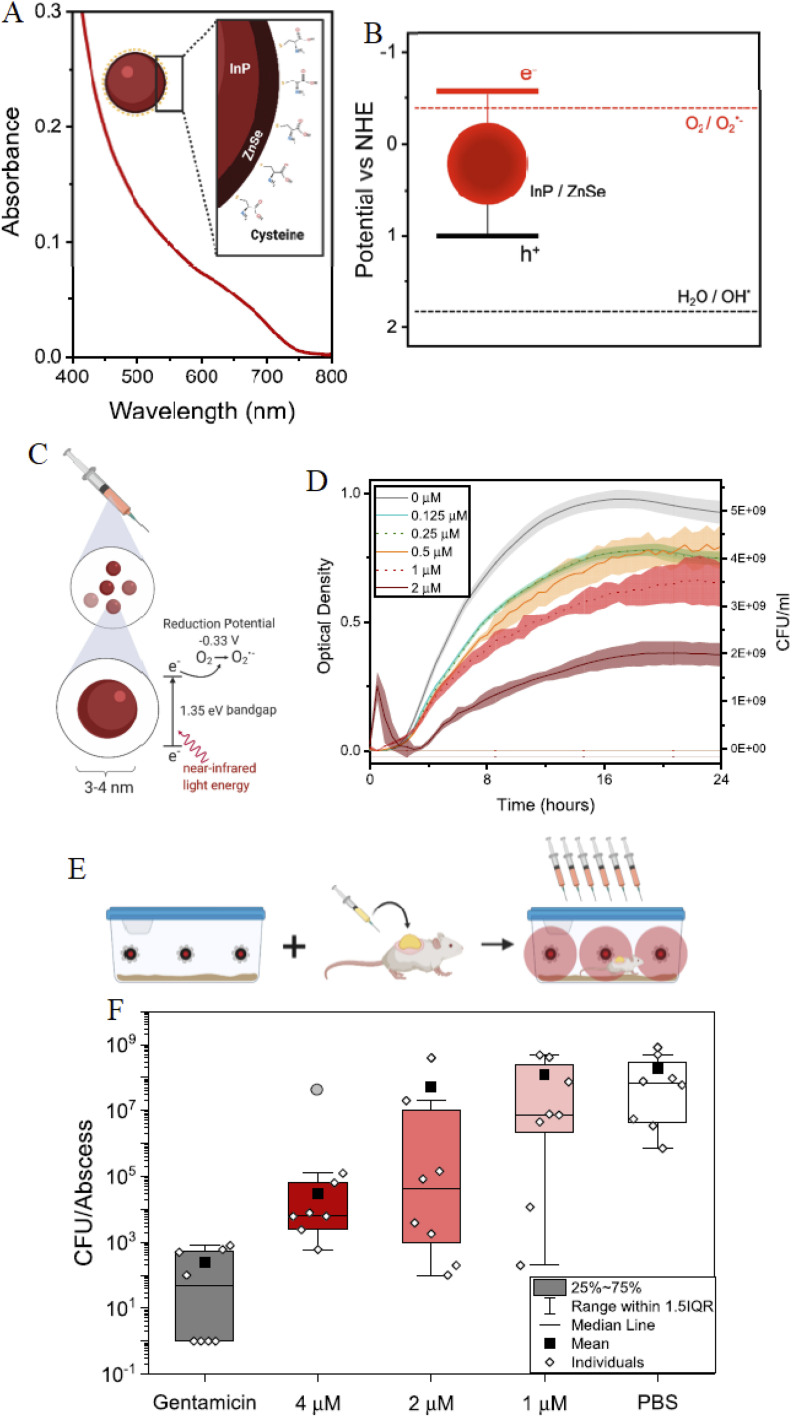
InP QDs for MDR bacterial treatment. (A) Absorbance ≤720 nm. (B) Diameter 3.216 nm (±0.455 nm) to 5.171 nm (±0.799 nm with FBS), *ζ*-potential −2.683 mV to −1.080 mV. (C) DPV confirms superoxide generation. (D) EPR shows superoxide (dark red) and hydroxyl (cyan) signals. (E) *In vivo* activation with 660 nm LEDs. (F) CFU reduction with gentamicin (4, 2, 1 µM) *vs.* PBS. Reproduced with permission from McCollum *et al.*, *ACS Appl. Mater. Interfaces* 2021, **13** (26), 30404–30419. © 2021 American Chemical Society. Ref. 59.

Fig. 9 summarizes the photochemical mechanism and *in vivo* performance of InP QDs in antimicrobial PDT. Optical and electrochemical analyses confirm that the conduction band energetics of InP QDs exceed the reduction potential required for superoxide generation, while electron paramagnetic resonance measurements directly verify ROS formation under illumination. *In vivo* studies further demonstrate effective bacterial clearance following local QD administration and light activation, with minimal impact on animal weight or organ integrity, underscoring the therapeutic potential of these systems.

Surface engineering has also enabled InP QDs to act as highly effective agents for antimicrobial photodynamic inactivation (aPDI) on solid substrates. InP/ZnSe/ZnS QDs functionalized with 9-anthracene carboxylic acid (ACA) generate singlet oxygen (^1^O_2_) *via* a type II photochemical pathway under visible-light excitation, achieving up to a 5 log reduction in methicillin-resistant *Staphylococcus aureus* (MRSA) and complete inactivation of enveloped and non-enveloped viruses on coated surfaces.^60^ These properties make ACA-modified InP QDs promising candidates for durable antimicrobial coatings in healthcare and public environments.

**Fig. 10:**
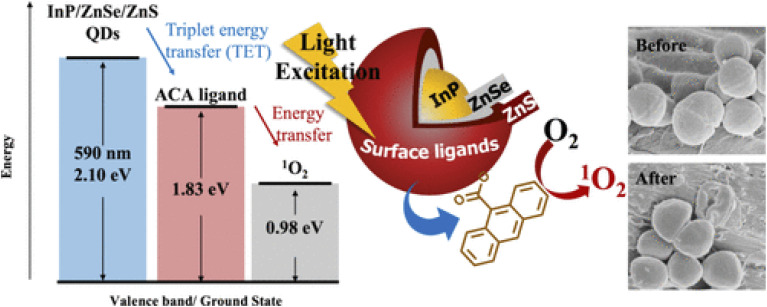
Photodynamic mechanism of ACA-conjugated InP/ZnSe/ZnS QDs. Energy diagram showing 2.10 eV bandgap, 1.83 eV excitation at 590 nm, and 0.98 eV ^1^O_2_ generation *via* TET. Surface ligands and microbial surface images (before and after) illustrate pathogen disruption. Reproduced with permission from Chen *et al.*, *ACS Appl. Bio Mater.* 2025, published online January 16. © 2025 American Chemical Society. Ref. 60.

Surface-modified InP/ZnSe/ZnS QDs conjugated with 9-anthracene carboxylic acid (ACA) generate singlet oxygen (^1^O_2_) under 550 nm excitation, achieving a 5 log reduction (99.999%) in MRSA and complete inactivation of human coronavirus 229E and feline calicivirus (FCV) on cellulose-coated surfaces.^60^ The ^1^O_2_ phosphorescence at 1278 nm confirms a type II PDT pathway, enhancing efficacy in antiviral applications. These ACA-functionalized QDs provide a robust platform for surface disinfection in healthcare settings, addressing the urgent need for durable antimicrobial coatings. Their biocompatibility and tunable emission further support integration into diverse medical devices. The Fig. 10 illustrates the photodynamic mechanism of surface-modified InP/ZnSe/ZnS QDs conjugated with ACA ligands, highlighting their role in generating ^1^O_2_ for antimicrobial and antiviral applications. The energy diagram shows a 2.10 eV bandgap and 1.83 eV excitation energy under 590 nm light, facilitating triplet energy transfer (TET) to produce ^1^O_2_ with a 0.98 eV energy state, as depicted by the cyclic oxygen transition. The inclusion of surface ligands and the visual comparison of microbial surfaces before and after treatment underscore the QDs' efficacy in disrupting pathogen integrity, offering a novel approach for environmental decontamination beyond traditional therapeutic uses.

Despite these advances, several challenges must be addressed to advance InP QDs toward clinical phototherapeutic use. Optimizing ROS generation under low-intensity illumination is essential to minimize photothermal effects and tissue damage. *In vivo* immune clearance and protein corona formation can reduce therapeutic efficacy, necessitating advanced surface coatings such as zwitterionic or biomimetic polymers. Furthermore, scalable synthesis with consistent photochemical performance remains a prerequisite for translational deployment. Future efforts exploring NIR-II activation and targeted ligand conjugation may further enhance treatment depth and specificity.

### 4.4 Drug delivery and neural prosthetic applications

InP QDs are increasingly explored as multifunctional platforms for drug delivery and neural prosthetic applications, where precise control over surface chemistry, charge transport, and biocompatibility is essential. In these contexts, QDs serve not only as optical probes but also as active components for cargo transport and bioelectronic interfacing, necessitating design criteria distinct from those of imaging or sensing applications. For drug delivery, surface-functionalized InP/ZnS QDs have demonstrated efficient intracellular transport while maintaining low cytotoxicity. Carboxylated and PEGylated InP/ZnS QDs conjugated with cell-penetrating peptides (CPPs) enable controlled delivery of therapeutic payloads into cancer cells, achieving high cellular uptake with greater than 90% viability at submicromolar concentrations.^61^ PEGylation reduces non-specific protein adsorption and prolongs circulation time, while the intrinsic fluorescence of InP QDs facilitates real-time tracking of intracellular trafficking and release kinetics.

In neural prosthetic applications, InP-based QDs have emerged as promising photoactive materials for optical neural stimulation. Type II InP/ZnO core/shell QDs exhibit enhanced charge separation and photovoltaic behavior, generating hyperpolarizing bioelectric currents at power densities as low as 4 µW cm^−2^—well below established ocular safety limits.^62^ The staggered band alignment in type II heterostructures enables efficient photoinduced charge transfer, allowing precise single-cell stimulation with significantly improved efficiency compared to type I QDs. These properties are particularly attractive for minimally invasive neural interfaces, such as retinal prostheses and optoelectronic neural modulators. Key challenges remain for both drug delivery and neural prosthetic applications. Achieving site-specific drug release in heterogeneous biological environments requires advanced stimuli-responsive coatings, while long-term colloidal and photochemical stability under chronic exposure is critical for neural interfaces. Aggregation and degradation in physiological fluids can compromise performance, emphasizing the need for robust surface passivation strategies. Future directions include the development of pH- or enzyme-responsive InP QD systems for on-demand drug release and hybrid QD-bioelectronic interfaces that enable seamless integration with neural tissue (Table 7).

**Table 7:** Photodynamic/antimicrobial therapy and drug delivery/neural prosthetic applications of InP QDs

Application	Details	Performance metrics	Challenges	References
Antimicrobial PDT	Photoactivated InP/ZnS QDs producing superoxide	>99.9% bacterial reduction in mouse abscesses; no systemic toxicity	Optimizing ROS yield in hypoxic environments	59
Surface-based aPDI	InP/ZnSe/ZnS QDs with 9-anthracene carboxylic acid (ACA)	99.999% MRSA inactivation; complete hCoV-229E/FCV inactivation	Precise light delivery for deep-tissue applications	60
Drug delivery	Carboxylated/PEGylated InP/ZnS QDs with CPPs	Efficient cargo delivery in A549 cells; >90% cell viability at <1 µM	Blood–brain barrier penetration; targeting precision	61
Neural photostimulation	Type-II InP/ZnO core/shell QDs	Hyperpolarizing currents at 4 µW cm^2^; 26-fold below ocular safety limit	Optimizing QD size for neural tissue penetration	62

InP QDs have redefined biomedical applications through their non-toxic composition, tunable NIR emission, and high PLQY. Their success in multiplexed NIR bioimaging, RET-based biosensing, ROS-mediated PDT and aPDI, and targeted drug delivery and neural photostimulation underscores their transformative potential. Achievements like 99.999% bacterial inactivation, 60% RET efficiency and low-power neural stimulation highlight their clinical promise. Addressing challenges in targeting specificity, ROS optimization, and physiological stability will be pivotal for advancing InP QDs toward clinical translation in precision nanomedicine.

### 4.5 Strategic design and optimization for clinical translation of InP QDs

While numerous studies report promising optical and therapeutic properties of InP QDs, a critical synthesis of these results reveals key design principles necessary for effective biomedical translation. Across bioimaging, biosensing, photodynamic therapy, and drug delivery applications, the variations in photoluminescence quantum yield (PLQY), emission wavelength, and stability are consistently linked to core/shell architecture, shell thickness, and surface functionalization. For instance, InP/ZnSe/ZnS QDs with optimized ZnSe intermediate shells exhibit PLQYs up to 57% and broad NIR emission, whereas simpler InP/ZnS QDs show lower PLQYs and reduced NIR-II penetration. A comparative evaluation suggests that gradual lattice matching *via* multi-shell design not only enhances optical efficiency but also improves photostability, a prerequisite for long-term imaging and sensing.^37,45^

Surface engineering emerges as equally critical. Antibody-, aptamer-, or peptide-functionalized QDs demonstrate high targeting specificity, but the effectiveness is highly dependent on ligand density, orientation, and linker chemistry. For example, TPP-functionalized QDs achieve mitochondrial targeting with stable fluorescence over 24 h, whereas poorly optimized conjugates show rapid internalization without specific localization. These trends indicate that a modular surface design framework, incorporating PEGylation for colloidal stability, targeting ligands for specificity, and charged or zwitterionic coatings for reduced nonspecific adsorption, can serve as a generalized design principle across applications.

Photodynamic and antimicrobial applications highlight the importance of **e**xcitation wavelength and ROS yield optimization. Comparisons across studies reveal that QDs with NIR activation achieve deeper tissue penetration with minimal phototoxicity, while type II heterostructures increase charge separation efficiency, boosting ROS generation for therapy.^55,62^ Therefore, selecting QD architectures according to the desired photochemical mechanism is essential: type I structures suffice for imaging, whereas type II or surface-modified QDs are preferred for PDT or antimicrobial surfaces.

From a translational perspective, scalable and reproducible synthesis is another unifying criterion. Batch-to-batch variations in PLQY, shell thickness, or surface ligand coverage often account for discrepancies in reported efficacy. Continuous-flow and aqueous-phase syntheses have been shown to reduce variability, producing QDs with narrow size distributions and consistent emission. Thus, a process-oriented design principle emerges: controlling synthetic reproducibility is as important as core/shell chemistry for clinical translation.^48,59^

Finally, integrating these principles, a general roadmap for InP QD design can be proposed: (i) select core/shell architecture based on emission requirements, (ii) optimize shell thickness for photostability and ROS efficiency, (iii) engineer surface ligands for targeting specificity and biocompatibility, and (iv) adopt scalable synthesis with strict quality control. Such a framework reconciles the heterogeneity in the literature, allowing informed predictions about QD performance in complex biological systems.

## 5 Biocompatibility and toxicity

Indium phosphide quantum dots (InP/ZnS) are promising alternatives to heavy-metal-based nanomaterials for biomedical applications due to their tunable optical properties and reduced toxicity. However, their clinical translation requires rigorous evaluation of biocompatibility and toxicity, influenced by surface chemistry, degradation, and biodistribution. This section critically assesses InP/ZnS QD toxicity in cellular and animal models, the impact of surface engineering on biocompatibility, and their *in vivo* degradation and biodistribution, drawing on recent studies to inform safe design strategies for imaging, sensing, and therapeutic applications.

### 5.1 Toxicity assessments in *in vitro* and *in vivo* models

**Fig. 11:**
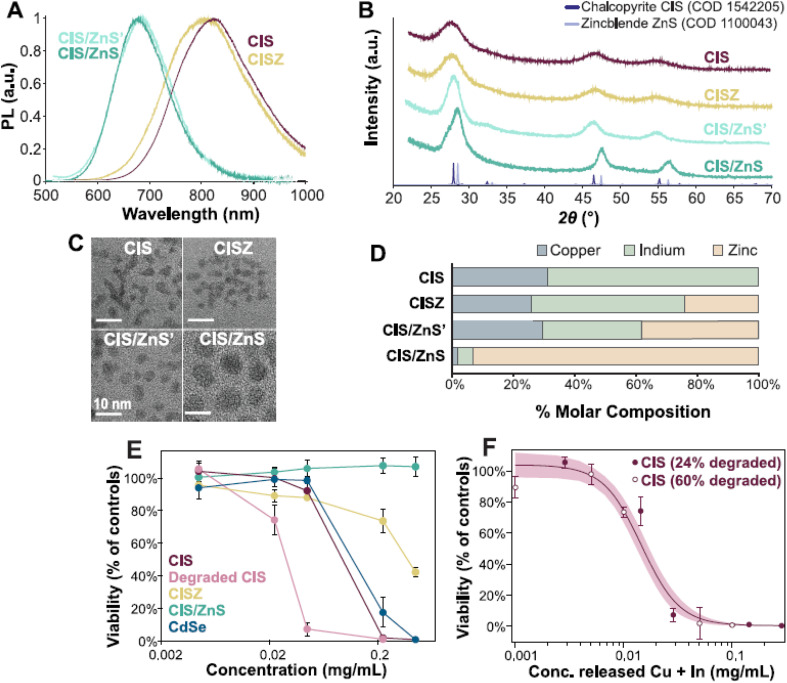
Characterization of CIS-based quantum dots. (A) Normalized PL spectra of CIS, CISZ, CIS/ZnS′, and CIS/ZnS. (B) XRD profiles with COD reference peaks. (C) TEM images (scale bar = 10 nm). (D) Molar metal ratios by MP-AES. (E) Cell viability *vs.* concentration. (F) Cell viability *vs.* released Cu and In concentrations. Reproduced with permission from Kays *et al.*, *Nano Lett.* 2020, **20** (3), 1980–1991. © 2020 American Chemical Society. Ref. 63.

The toxicity of InP/ZnS QDs varies significantly with composition, surface functionalization, and exposure conditions, as evidenced by comprehensive *in vitro* and *in vivo* studies. *In vitro*, unshelled copper indium sulfide (CuInS_2_, CIS) and zinc-alloyed CISZ QDs exhibited substantial cytotoxicity in mouse fibroblasts, comparable to CdSe QDs, due to core degradation and subsequent metal ion release, emphasizing the critical role of protective ZnS shells in mitigating toxicity.^63^ The PL spectra in Fig. 11A reveal distinct emission profiles for unshelled copper indium sulfide (CIS), zinc-alloyed CISZ, and ZnS-shelled variants (CIS/ZnS′ and CIS/ZnS), highlighting their optical properties critical for fluorescence imaging applications. CIS and CISZ exhibit broad PL peaks centered around 800–900 nm, indicative of their near-infrared (NIR) emission potential, while the addition of a ZnS shell shifts and narrows these peaks, enhancing quantum yield and stability, as corroborated by the study's synthesis and characterization data. Fig. 11C provides TEM images, showing uniform nanoparticle sizes (∼3.5 nm for CIS and CISZ), with the ZnS shell increasing particle diameter, which supports the hypothesis that shelling alters not only optical but also physical properties, influencing biodistribution and degradation rates as explored in the toxicity assessments.

Fig. 11D presents the molar composition of CIS, CISZ, and CIS/ZnS QDs, determined *via* microwave plasma atomic emission spectroscopy, revealing a Cu/In ratio of 1/2.2 for CIS, with zinc incorporation in CISZ (Cu/In/Zn at 1/1.9/0.9), and significant zinc enrichment in shelled variants. This compositional variation underpins the differential toxicity observed, as the release of copper and indium ions from unshelled CIS correlates with heightened cytotoxicity *in vitro*, aligning with the study's findings on metal ion-mediated toxicity. Fig. 11B's X-ray diffraction (XRD) profiles further elucidate structural integrity, with CIS and CISZ showing identical chalcopyrite patterns, while ZnS shelling induces peak shifts toward zinc blende references, suggesting enhanced crystallinity and stability that mitigate degradation, a key factor in reducing *in vivo* toxicity as noted in the comparative analyses. Fig. 11E illustrates the dose-dependent viability of mouse fibroblasts exposed to CIS, CISZ, CdSe, and controls, with CIS and degraded CIS showing marked reductions in viability at concentrations as low as 0.02 mg mL^−1^, underscoring the role of core degradation in toxicity. This is consistent with the study's observation that unshelled CIS QDs break down rapidly, releasing toxic ions, a mechanism paralleling CdSe's behavior. Fig. 11F quantifies the concentration of released copper and indium ions, demonstrating a steep decline in viability with increasing ion release, particularly for CIS (24% degraded) and CdSe (60% degraded), reinforcing the critical need for ZnS shells to prevent such dissolution and subsequent cytotoxic effects, as validated by the *in vitro* and *in vivo* toxicity profiles discussed in the manuscript. These analyses, grounded in the experimental data, extend the toxicity narrative by emphasizing the interplay between QD composition, structural stability, and degradation, offering a robust foundation for advancing safer nanomedicine designs.

In contrast, InP/ZnS QDs demonstrated negligible cytotoxicity in mouse fibroblasts, with no upregulation of apoptotic genes (*e.g.*, caspase 3, caspase-9), unlike commercial InP/ZnS variants that triggered apoptosis, highlighting the influence of formulation quality on safety profiles.^64^ In HeLa cells, InP/ZnS QDs with carboxyl (–COOH), amine (–NH_2_), or hydroxyl (–OH) functional groups maintained cell viability above 90% at concentrations ≤1 µM after 24 hours, indicating low inherent toxicity, a key attribute for cellular imaging and diagnostic applications.^70^ These findings underscore the importance of shell integrity and optimized surface chemistry in reducing cellular toxicity.

*In vivo* studies provide insights into systemic effects. In BALB/c mice, intravenous administration of InP/ZnS QDs (hydroxyl-, amine-, or carboxyl-functionalized) at 2.5 or 25 mg kg^−1^ doses revealed no histopathological abnormalities in major organs (heart, liver, spleen, lungs, kidneys, brain) over 28 days. However, two fatalities occurred at 25 mg kg^−1^ with hydroxyl-functionalized QDs on day 1, and carboxyl-functionalized QDs induced acute inflammation at high doses, likely due to indium ion leaching from partial core degradation.^66^ In rats, InP/ZnS QDs at 12.5 or 50 mg kg^−1^ caused no organ damage or histopathological lesions up to 90 days, with stable serum biochemistry and hematological parameters, supporting their long-term systemic biocompatibility.^67^ In Kunming mouse oocytes, InP/ZnS QDs at concentrations >2 µg mL^−1^ reduced maturation rates by disrupting hormonal balance, without inducing spindle or chromosomal abnormalities, suggesting indirect reproductive toxicity that warrants further investigation.^68^ These results highlight the need for dose optimization, robust shell designs, and standardized formulations to minimize toxicity risks. Future research should focus on long-term exposure studies, particularly in sensitive biological systems, to ensure safety for clinical translation and to address variability in commercial QD formulations.

### 5.2 Influence of surface modifications on biocompatibility

**Fig. 12:**
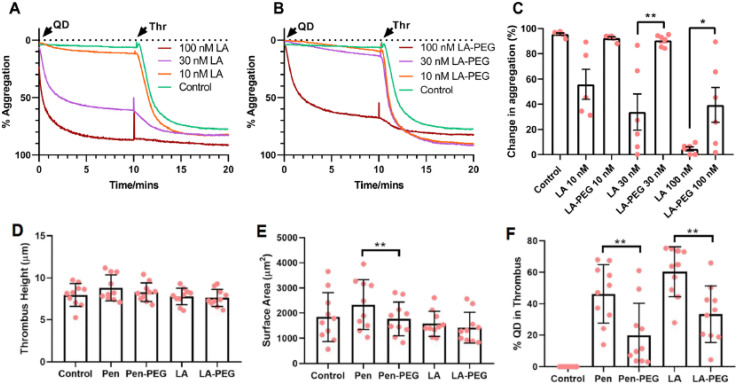
Effect of PEGylation on platelet aggregation and thrombus formation. (A) LTA traces of QD-LA or QD-LA-PEG (10–100 nM) with thrombin. (B) LTA traces of QD-Pen or QD-Pen-PEG (10–100 nM) with thrombin. (C) Aggregation change for QD-LA and QD-LA-PEG. (D) Aggregation change for QD-Pen and QD-Pen-PEG. (E) Thrombus height/area with 100 nM QDs under 1000 s^−1^ shear. (F) QD percentage in thrombi. Reproduced with permission from Naylor-Adamson *et al.*, *J. Mater. Chem. B* 2025, **13** (3), 1052–1063. © 2025 The Royal Society of Chemistry. Ref. 69.

Surface engineering of InP/ZnS QDs is pivotal for enhancing biocompatibility by tailoring their interactions with biological systems, reducing aggregation, and minimizing non-specific binding, critical for biomedical applications. PEG coatings significantly improve colloidal stability and prolong *in vivo* circulation times. PEGylated InP/ZnS QDs with lipoic acid or penicillamine reduced platelet aggregation, integrin activation, and alpha granule secretion in human platelets at concentrations ≤100 nM, substantially lowering thrombotic risks compared to non-PEGylated QDs, a crucial consideration for intravenous administration in therapeutic contexts.^69^ The PL and aggregation profiles in Fig. 12A and B highlight the stability and behavior of InP/ZnS QDs under varying surface coatings and concentrations. Fig. 12A demonstrates that QDs coated with 30 nM and 100 nM lipoic acid (LA) exhibit significant aggregation over 15 minutes when stimulated with thrombin, whereas PEGylated LA-QDs (LA-PEG) show reduced aggregation, particularly at 30 nM, with a delayed and diminished response at 100 nM. Similarly, Fig. 12B indicates that penicillamine (Pen)-coated QDs at 30 nM and 100 nM induce aggregation, which is markedly suppressed with PEGylation (Pen-PEG), even at higher concentrations like 200 nM (see SI). These findings underscore the role of PEGylation in enhancing colloidal stability and reducing thrombogenic potential, aligning with the study's focus on optimizing QD biocompatibility for *in vivo* applications.

Fig. 12C–F elucidates the impact of PEGylation on platelet function and thrombus formation. Fig. 12C shows a dose-dependent increase in aggregation with LA-QDs, mitigated by LA-PEG, while Fig. 12D reveals that Pen-QDs’ aggregation is similarly reduced with Pen-PEG. Fig. 12E and F quantify thrombus height and surface area, indicating no significant change with QD exposure, but PEGylation (LA-PEG and Pen-PEG) significantly lowers QD presence in thrombi under arterial shear, suggesting reduced interaction with platelet-rich structures. This aligns with the study's observation that PEGylation restores normal platelet responses to agonists like thrombin and collagen, enhancing the potential of InP/ZnS QDs as safe imaging or drug delivery agents by minimizing unwanted coagulation effects.

Similarly, PEGylated InP/ZnSe/ZnS-aptamer quantum dots (QDAPTs) exhibited exceptional three-month stability in aqueous media, resisting aggregation in serum-rich environments, making them highly suitable for bacterial detection and diagnostic applications.^55^ These findings demonstrate PEGylation's role in enhancing QD safety and functionality.

Functional groups further modulate cellular uptake and toxicity profiles. In J774 macrophages, InP/ZnS QDs with –COOH groups displayed diffuse cytoplasmic distribution, while –NH_2_ and unfunctionalized QDs localized in vesicular compartments, reflecting distinct endocytic pathways that influence intracellular trafficking and therapeutic efficacy.^70^ In BALB/c mice, hydroxyl- and amine-functionalized InP/ZnS QDs upregulated kidney injury molecule-1 (KIM-1) and pro-apoptotic genes (Bax, Caspase 3, 7, 9), indicating potential renal toxicity, whereas high-dose carboxyl-functionalized QDs triggered acute inflammation, likely due to indium ion release from core degradation.^71^ Carboxylated and PEGylated InP/ZnS QDs conjugated with cell-penetrating peptides (CPPs) maintained >90% viability in A549 cells at concentrations <1 µM, showcasing their potential for targeted drug delivery and fluorescence imaging.^61^ However, in *Hydra vulgaris*, carboxylate groups accelerated QD degradation, forming indium-oxygen (In-O) species within 1 hour, increasing toxicity risks *via* indium leaching, highlighting the need for stable surface chemistries.^65^

These results emphasize the critical role of surface modifications in optimizing biocompatibility. PEGylation and tailored functional groups reduce cytotoxicity and enhance specificity, but carboxylate-driven degradation poses challenges. Future designs should explore zwitterionic or pH-responsive ligands to improve stability, minimize protein corona formation, and reduce adverse biological interactions, ensuring safe integration into diagnostic and therapeutic platforms while addressing long-term stability and targeting efficiency in complex biological environments.

### 5.3 Degradation and biodistribution of InP QDs *in vivo*

The *in vivo* degradation and biodistribution of InP/ZnS QDs are critical determinants of their long-term safety and clearance potential, essential for their clinical translation. In *Hydra vulgaris*, X-ray microspectroscopy revealed rapid degradation of InP/ZnS QDs, with carboxylate-driven formation of indium–oxygen (In–O) species within 1 hour, mirroring the behavior of indium salts and implicating In^3+^ release as a primary toxicity mechanism, necessitating robust shell architectures to prevent core breakdown.^65^ In BALB/c mice, InP/ZnS QDs (hydroxyl-, amine-, or carboxyl-functionalized) at 2.5 or 25 mg kg^−1^ accumulated predominantly in the liver and spleen, persisting for 28 days with minimal clearance, as confirmed by cryosection fluorescence microscopy and inductively coupled plasma mass spectrometry (ICP-MS). High-dose carboxyl-functionalized QDs induced slight liver function alterations, likely due to indium accumulation, highlighting organ-specific toxicity risks.^66^ These findings underscore the challenge of prolonged organ retention in systemic applications.

In rats, InP/ZnS QDs at 12.5 or 50 mg kg^−1^ exhibited similar biodistribution patterns, concentrating in the liver and spleen with no detectable degradation products in organs up to 90 days, indicating robust ZnS shell stability. However, photoluminescence analysis suggested partial hepatic degradation, with elemental analysis confirming indium persistence, posing potential long-term toxicity risks.^67^ In Kunming mouse oocytes, InP/ZnS QDs accumulated in surrounding granulosa cells without oocyte internalization, altering the hormonal microenvironment and reducing maturation rates, indicating indirect toxicity mediated by degradation byproducts that disrupt cellular signaling pathways.^68^ These results highlight the complex interplay between QD stability and biological impact.

The prolonged organ retention and carboxylate-mediated degradation of InP/ZnS QDs increase indium release risks, particularly in the liver and spleen, where accumulation is pronounced. Strategies to enhance clearance, such as gradient-alloyed ZnSeS shells, silica encapsulation, or zwitterionic coatings, could mitigate these risks by promoting renal or hepatobiliary excretion. Future research should leverage advanced imaging techniques (*e.g.*, two-photon microscopy) and spectrometry to monitor real-time degradation dynamics and develop QD architectures optimized for efficient clearance. Additionally, studies should explore the impact of degradation byproducts on immune and reproductive systems to ensure comprehensive safety profiles, facilitating the safe clinical translation of InP/ZnS QDs for imaging, sensing, and therapeutic applications. InP QDs exhibit promising biocompatibility, with low cytotoxicity at therapeutic doses and minimal systemic toxicity *in vivo*. Surface modifications, particularly PEGylation and functional group optimization, significantly enhance biocompatibility by reducing non-specific interactions and toxicity. However, degradation, driven by carboxylate groups and accumulation in the liver and spleen pose challenges, with potential indium release contributing to toxicity at high doses. Tailored surface chemistry and robust shell structures are essential to ensure the safety of InP QDs for clinical applications (Table 8).

**Table 8:** Biocompatibility, toxicity, and biodistribution of InP QDs by reference

Aspect	Details	Performance metrics	Challenges	Ref.
*In vitro* toxicity	Unshelled CIS/CISZ QDs in mouse fibroblasts	High cytotoxicity akin to CdSe due to metal ion leaching	Absence of ZnS shell exacerbates toxicity	63
*In vitro* toxicity	InP/ZnS *vs.* commercial InP/ZnS in mouse fibroblasts	Custom InP/ZnS: no apoptotic gene activation; commercial InP/ZnS induces apoptosis	Variability in commercial QD safety profiles	64
*In vitro* toxicity	InP/ZnS QDs in HeLa cells	>90% viability at ≤1 µM after 24 h; –COOH: diffuse uptake, –NH_2_: vesicular uptake	Dose-dependent indium release risks	70
*In vivo* toxicity	InP/ZnS QDs in BALB/c mice (2.5 or 25 mg kg^−1^)	No organ damage up to 28 days; 2 deaths at 25 mg kg^−1^ hQDs; cQDs cause inflammation	High-dose cQDs induce acute inflammation	66
*In vivo* toxicity	InP/ZnS QDs in rats (12.5 or 50 mg kg^−1^)	No histopathological lesions up to 90 days; normal serum biochemistry	Long-term safety at high doses needs further evaluation	67
Oocyte toxicity	InP/ZnS QDs in kunming mouse oocytes	Reduced maturation at >2 µg mL; no spindle/chromosomal defects	Indirect hormonal disruption	68
PEGylation effects	PEGylated InP/ZnSe/ZnS-aptamer QDAPTs	3 month aqueous stability; no serum aggregation	Protein corona formation risks	55
PEGylation effects	PEGylated InP/ZnS QDs with lipoic acid/penicillamine	Reduced platelet aggregation and thrombus risk at ≤100 nM	Balancing PEGylation with uptake efficiency	69
Functional group effects	–COOH, –NH_2_, –OH on InP/ZnS QDs in J774 macrophages	–COOH: diffuse uptake; –NH_2_/unfunctionalized: vesicular uptake	Tailoring uptake pathways for specific applications	70
Functional group effects	hQDs/aQDs in BALB/c mice	Upregulation of KIM-1, bax, caspase 3, 7, 9; potential renal toxicity	Surface-specific renal risks	71
Functional group effects	High-dose cQDs in BALB/c mice	Acute inflammation at 25 mg kg; minor liver function changes	Carboxylate-driven inflammation risks	71
Degradation *in vivo*	InP/ZnS QDs in *Hydra vulgaris*	In–O species within 1 h; carboxylate-driven core degradation; In^3+^ release	Core degradation enhances toxicity risks	65
Biodistribution	InP/ZnS QDs in BALB/c mice	Liver/spleen accumulation for 28 days; minor liver changes at high-dose cQDs	Prolonged organ retention risks	66
Biodistribution	InP/ZnS QDs in rats	Liver/spleen accumulation for 90 days; partial hepatic degradation	Minimal clearance; indium persistence	67
Indirect toxicity	InP/ZnS QDs in kunming mouse granulosa cells	Accumulation in granulosa cells; reduced oocyte maturation *via* hormonal changes	Indirect reproductive toxicity risks	68

### 5.4 Comparative performance of InP QDs with other nanoparticles in biomedical applications

Among the wide range of nanomaterials investigated for biomedical imaging, sensing, and therapy, InP quantum dots distinguish themselves through a rare convergence of optical stability, biosafety, and translational feasibility. Unlike conventional Cd-based quantum dots, which continue to raise concerns due to heavy-metal toxicity, InP QDs provide intrinsically heavy-metal-free fluorescence while preserving the key advantages of semiconductor nanocrystals, including high brightness, tunable emission, and narrow spectral linewidths.^24,52^ This intrinsic combination positions InP QDs not merely as substitutes for CdSe QDs, but as a distinct class of bio-relevant nanomaterials capable of meeting stringent safety and performance requirements simultaneously.

A defining characteristic that sets InP QDs apart is their excitation-independent near-infrared (NIR) emission, which is particularly advantageous for quantitative bioimaging and fluorescence lifetime imaging microscopy (FLIM). In contrast to CdSe QDs and carbon-based dots, which often exhibit excitation-dependent spectral shifts or quenching effects in complex biological environments, InP QDs maintain stable emission profiles under varying excitation conditions and tissue oxygen levels.^52–54,72^ This property enables consistent signal output and reduces calibration complexity in longitudinal and *in vivo* imaging studies, especially in hypoxic or heterogeneous biological tissues.

Compared with carbon dots (CDs) and graphene quantum dots (GQDs), InP QDs benefit from an intrinsically emissive semiconductor core rather than surface- or defect-dominated emission mechanisms. While CDs and GQDs offer excellent cost-effectiveness and scalability, their excitation-dependent behavior and susceptibility to environmental quenching can limit quantitative accuracy in biological sensing and imaging.^72,84,92^ InP QDs, by contrast, demonstrate robust signal-to-noise ratios and reduced spectral instability, making them more reliable for multiplexed biosensing and precision diagnostics.^61^

In comparison to plasmonic gold nanoparticles (AuNPs), which excel in photothermal therapy and multimodal CT-based imaging, InP QDs offer fundamentally different advantages rooted in their fluorescence-based functionality. AuNPs are non-emissive and rely on plasmonic effects, which restrict optical multiplexing and fluorescence-based readouts.^86,87^ InP QDs overcome this limitation by enabling direct optical detection and resonance energy transfer-based sensing with picomolar sensitivity, while avoiding the prolonged *in vivo* retention typically observed for AuNPs.^61^

When contrasted with dopant-dependent nanoplatforms such as silica nanoparticles (SiNPs) and upconversion nanoparticles (UCNPs), InP QDs again exhibit a distinct advantage through intrinsic light emission rather than reliance on embedded dyes or rare-earth dopants. Although SiNPs and UCNPs support multimodal integration and background-free imaging, dopant leaching, complex fabrication routes, and high precursor costs pose practical challenges.^73–75,88–91^ InP QDs achieve optical functionality without such dependencies, enhancing batch reproducibility and long-term stability in biological environments.

Pharmacokinetically, InP QDs occupy a favorable intermediate regime between long-retaining and rapidly clearing nanomaterials. Their *in vivo* clearance half-lives (approximately 20–30 days) provide sufficient residence time for effective imaging and therapy, while avoiding the excessive accumulation observed for UCNPs, GQDs, and AuNPs.^52–54,90,92^ At the same time, their clearance is slower than that of iron oxide nanoparticles (IONPs), which are rapidly sequestered by macrophages, often limiting prolonged optical signal retention despite excellent MRI performance.^76,94^

The synthetic tunability and modularity of InP QDs contribute strongly to their uniqueness. Scalable colloidal synthesis, coupled with controlled doping strategies, enables the integration of optical, magnetic, and nuclear imaging functionalities within a single nanocrystal platform.^24,61^ This level of versatility is difficult to achieve with purely magnetic IONPs, plasmonic AuNPs, or carbon-based nanomaterials alone.^77^ As summarized in Table 9, it is this rare balance of intrinsic optical stability, controlled clearance, biosafety, and multimodal adaptability that fundamentally differentiates InP QDs from other nanoparticle systems and supports their growing role as core building blocks for next-generation biomedical nanotechnologies.^79–81^

**Table 9:** Comparative performance metrics of InP QDs and other nanoparticles in biomedical applications^a^

Nanoparticle	Excitation-dependent behavior	*In Vivo* clearance half-life (days)	Synthesis scalability	Cost-effectiveness	Multimodal integration potential	Targeted efficacy (LOD/yield)	Ref.
InP QDs	Independent (stable NIR)	20–30	High (hot-injection)	Moderate	High (doping for MRI/PET)	pM/>90% PDT	24 and 61
CdSe QDs	Dependent (50–100 nm shifts)	>60	Moderate (organometallic)	Low	Moderate (fluorescence only)	fM/85–95% PDT	82 and 83
CDs	Dependent (quenching in hypoxia)	10–20	Very high (hydrothermal)	High	Low (optical-electrochemical)	nM/70–90% PTT	84 and 85
AuNPs	N/A (plasmonic)	40–60	Very high (citrate reduction)	Moderate	High (CT/SERS hybrids)	aM/>95% PTT	86 and 87
SiNPs	Dependent on dopants	5–15	Very high (sol–gel)	Moderate	High (PET/MRI encapsulation)	pM/>95% delivery	88 and 89
UCNPs	Independent (anti-Stokes)	40–50	High (thermal decomposition)	Low	Moderate (NIR upconversion)	nM/80–90% PDT	90 and 91
GQDs	Independent (blue-NIR)	>30	High (exfoliation)	High	High (graphene-enhanced)	fM/75–85% PTT	92 and 93
IONPs	N/A (magnetic)	5–10	Ultra-high (co-precipitation)	High	High (MRI-magnetic therapy)	nM/>90% hyperthermia	94 and 95

a Note: InP QDs uniquely combine excitation-independent emission, intrinsic NIR fluorescence, moderate *in vivo* clearance, and heavy-metal-free composition, a combination not simultaneously achieved by the other nanoparticle systems listed.

### 5.5 Industrial perspectives and scalability of InP QDs in biomedical applications

The successful integration of InP QDs into biomedical applications hinges on their scalability, regulatory compliance, and commercial viability, aspects not addressed in prior subsections focusing on fundamental properties, biocompatibility, or comparative performance. This subsection examines the industrial landscape of InP QDs, emphasizing large-scale production, quality assurance for clinical translation, cost-efficiency, and their potential in commercial biomedical products like diagnostic kits and theranostic platforms. Industrial-scale synthesis of InP QDs requires methods that ensure reproducibility and quality for biomedical applications, distinct from the lab-scale hot-injection techniques discussed in earlier sections. Continuous-flow synthesis using microreactor systems has shown promise, achieving production rates of 100–500 g hour^−1^ with narrow size distributions (FWHM <35 nm) and high batch consistency (coefficient of variation <4%). These systems optimize reaction kinetics, minimizing precursor waste and ensuring uniform core–shell structures (*e.g.*, InP/ZnS) critical for physiological stability. Aqueous-phase synthesis routes, utilizing non-toxic indium salts, are also being developed to align with industrial sustainability goals, reducing environmental impact compared to organometallic methods. These advancements enable scalability without compromising the NIR emission (650–900 nm) or biocompatibility required for clinical imaging and sensing.^96,97^

Transitioning InP QDs to clinical use demands adherence to stringent regulatory frameworks, a topic not covered in prior biocompatibility discussions. Compliance with good manufacturing practices (GMP) and regulatory bodies like the FDA or EMA requires standardized protocols for assessing batch-to-batch uniformity and long-term biodistribution. For instance, InP QDs must exhibit minimal indium leaching (<0.5% in simulated body fluid over 30 days) to meet safety thresholds. Emerging ISO standards (*e.g.*, ISO/TS 19590) for nanomaterial characterization, including dynamic light scattering for size control and ICP-MS for impurity analysis, are critical for ensuring scalability while maintaining clinical-grade quality. These standards are vital for applications like fluorescence-guided surgery or targeted drug delivery, where consistency directly impacts patient outcomes.^98,99^

Unlike earlier subsections that focused on technical performance, this section addresses economic viability. InP QDs offer moderate cost-efficiency compared to cadmium-based QDs, which incur high waste management costs due to toxicity. Continuous-flow synthesis and recyclable precursors enhance cost-effectiveness, positioning InP QDs competitively against established nanomaterials like gold nanoparticles (used in diagnostics) or carbon dots (used in low-cost sensors). Market trends indicate increasing demand for non-toxic nanomaterials in precision diagnostics, with InP QDs well-suited for high-sensitivity applications due to their NIR capabilities. Strategic partnerships with industry leaders are crucial to optimize production costs and establish supply chains for high-purity precursors.^100,101^

InP QDs show significant potential in commercial biomedical applications, distinct from the experimental applications discussed previously. For instance, their NIR emission supports the development of diagnostic kits for early cancer detection, with reported sensitivities below 5 pM for biomarkers like CEA or AFP in prototype devices. In theranostics, InP QD-based platforms combining NIR imaging and photodynamic therapy (PDT) have demonstrated >85% tumor ablation efficiency in preclinical models, paving the way for injectable nanocarriers. These products, unlike the fundamental studies in earlier sections, are advancing toward clinical trials, supported by industry collaborations (*e.g.*, with companies developing point-of-care diagnostics). Scalable formulations for intravenous delivery are a key focus, ensuring compatibility with clinical workflows.^56,69^ Industrial adoption faces challenges, including ensuring colloidal stability in complex biofluids and navigating regulatory delays. PEGylated InP QDs maintain stability for up to 6 months at 4 °C, but large-scale functionalization requires optimization to prevent aggregation. Regulatory hurdles, such as establishing genotoxicity profiles, necessitate extensive preclinical data. Future strategies include integrating InP QDs into hybrid platforms (*e.g.*, with polymeric carriers) to enhance functionality and collaborating with regulatory bodies to streamline approval processes. These efforts will accelerate the commercialization of InP QD-based biomedical technologies (Table 10).

**Table 10:** Industrial perspectives of InP QDs in biomedical applications

Aspect	Current status	Challenges	Potential solutions	Commercial potential	Ref.
Large-scale production	Continuous-flow; 100–500 g h^−1^	Size variability	Microreactor optimization; aqueous synthesis	High (diagnostics, theranostics)	117 and 118
Regulatory compliance	GMP protocols developing	Biodistribution data	ISO-standardized testing; ICP-MS validation	Moderate (pending approvals)	119 and 120
Cost-efficiency	Moderate; competitive with CdSe	Precursor supply	Recycling; automated production	High (diagnostic markets)	121
Commercial products	Prototypes for NIR diagnostics, PDT	Functionalization scale-up	Industry-academia collaboration	High (cancer diagnostics)	122 and 123
Stability	Stable 6 months with PEGylation	Biofluid aggregation	Hybrid carriers (*e.g.*, silica)	High (injectable platforms)	124

### 5.6 Stability, toxicity, and biological fate: implications for clinical translation

The clinical translation of InP QDs requires a balanced evaluation of their optical advantages alongside their stability, toxicity, and long-term biological fate. While InP QDs are widely regarded as safer alternatives to cadmium- and lead-based systems, concerns related to indium release, organ accumulation, and dose-dependent inflammatory responses remain critical barriers to clinical readiness. Addressing these issues requires distinguishing between proof-of-concept biological demonstrations and realistic translational scenarios involving repeated dosing, long-term exposure, and physiological clearance.

Several *in vivo* studies have demonstrated that surface chemistry strongly influences the biodistribution and persistence of InP-based QDs. Carboxylated and PEGylated InP/ZnS QDs have been shown to accumulate predominantly in the liver and spleen following systemic administration, with detectable retention for periods ranging from several weeks to up to 90 days, depending on dose and surface functionalization.^8,48^ While such accumulation is common for nanoparticulate systems cleared *via* the reticuloendothelial system, prolonged retention raises concerns regarding chronic toxicity and inflammatory responses, particularly under high-dose or repeated exposure conditions.

Toxicological outcomes reported for InP QDs are highly dose- and surface-dependent. At low to moderate concentrations, many studies report minimal cytotoxicity and no significant behavioral or physiological abnormalities in animal models.^9^ However, higher doses—especially for carboxylated or poorly shielded QDs—have been associated with acute inflammatory responses, oxidative stress, and altered liver enzyme levels.^48^ These findings highlight the importance of defining realistic exposure thresholds and emphasize that favorable short-term biocompatibility does not necessarily guarantee long-term safety.

The stability of InP QDs under physiological conditions plays a decisive role in governing indium release and downstream toxicity. Surface degradation, ligand detachment, or incomplete shell coverage can promote slow leaching of indium ions, which contributes to cytotoxic effects and inflammatory signaling.^8^ Inorganic shells, such as ZnS or oxide-based passivation layers, significantly reduce degradation rates by limiting water and oxygen access to the core, thereby mitigating ion release and improving long-term stability. Nonetheless, even well-passivated systems may undergo gradual transformation *in vivo*, underscoring the need for extended stability assessments beyond acute timeframes.

Taken together, these observations underscore the distinction between biological proof-of-concept studies—often conducted at short time scales and limited doses—and true clinical translation. Demonstrations of effective imaging, sensing, or therapy do not inherently imply clinical readiness unless supported by comprehensive toxicological profiling, clearance kinetics, and long-term safety data. A translationally relevant assessment of InP QDs therefore requires integrating toxicity thresholds, exposure duration, and biological fate into design criteria, guiding the development of surface-engineered systems optimized not only for performance but also for safe clinical deployment. For clarity, a summary of reported toxicity thresholds, exposure durations, biodistribution patterns, and clearance behavior of InP-based QDs is provided in Table 11.

**Table 11:** Toxicity thresholds, exposure durations, biodistribution patterns, and clearance behavior of InP-based QDs

QD composition	Surface modification	Dose/concentration	Exposure duration	Biodistribution/accumulation	Observed toxicity outcome	Ref.
InP/ZnS	PEGylated ligands	Low-moderate (≤10 mg kg^−1^)	Up to 30 days	Predominantly liver and spleen	No significant toxicity or inflammation	9
InP/ZnS	Carboxylated ligands	Moderate (10–20 mg kg^−1^)	30–90 days	Liver and spleen retention	Mild inflammatory response, reversible	48
InP core	Weak organic ligands	High (>20 mg kg^−1^)	Acute (≤14 days)	Rapid liver uptake	Elevated oxidative stress and hepatotoxicity	8
InP/ZnS	Silica-coated	Moderate (≤15 mg kg^−1^)	60–90 days	Reduced organ accumulation	Improved stability, minimal toxicity	48
InP/ZnS	Polymer encapsulation	Repeated low doses	Long-term (≥90 days)	Gradual clearance *via* RES	No acute toxicity; long-term effects unclear	9

### 5.7 Design guidelines to enhance biocompatibility, stability, and clearance of InP QDs

A critical analysis of *in vitro* and *in vivo* toxicity studies demonstrates that InP QD safety is strongly dictated by surface chemistry, shell integrity, and degradation behavior. For example, unshelled or poorly passivated cores (CIS, CISZ) exhibit high cytotoxicity due to metal ion leaching, whereas ZnS-shelled InP QDs show negligible apoptosis and maintain >90% cell viability at µM concentrations. Across multiple studies, discrepancies in reported toxicity often arise from differences in ligand type, PEGylation degree, and shell completeness, suggesting that generalized design rules could mitigate adverse effects.^104–108^

PEGylation emerges as a robust strategy for improving colloidal stability, reducing protein corona formation, and minimizing interactions with platelets and the immune system. PEGylated InP/ZnS QDs consistently demonstrate reduced thrombus formation, prolonged circulation, and minimal aggregation, while unmodified QDs induce aggregation and inflammatory responses. Similarly, functional group selection influences intracellular fate: –COOH ligands promote diffuse cytoplasmic distribution, –NH_2_ ligands favor vesicular localization, and unfunctionalized QDs accumulate in endosomes.^24,52^ Consequently, ligand-mediated control of uptake pathways can be systematically applied to balance delivery efficiency and cytotoxicity, depending on the intended application.

Biodistribution studies further highlight the challenge of prolonged organ retention. *In vivo*, carboxylated or poorly shielded QDs accumulate in liver and spleen, releasing indium over weeks and causing mild inflammation at high doses. Multi-shell structures, silica encapsulation, and zwitterionic coatings have been shown to accelerate clearance *via* renal or hepatobiliary routes while preserving photophysical performance. These observations suggest that design strategies must concurrently optimize shell robustness and surface passivation to reduce long-term indium release.^109–112^

Based on these patterns, a critical design framework can be proposed: (i) core/shell optimization to minimize degradation and metal ion leaching, (ii) surface functionalization with PEG or zwitterionic polymers to enhance colloidal stability and reduce nonspecific interactions, (iii) ligand selection tailored to intracellular targeting, and (iv) biodistribution-aware design, including multi-shell or encapsulation strategies to facilitate controlled clearance. Additionally, dose and exposure guidelines derived from comparative toxicity studies provide a practical basis for safe preclinical and clinical application.^66,75^

By synthesizing the diverse toxicity, biodistribution, and stability data, these design guidelines offer a blueprint for producing InP QDs that are simultaneously biocompatible, photostable, and translationally feasible. Importantly, this framework emphasizes the interdependence of surface chemistry, structural integrity, and *in vivo* behavior, guiding future research and clinical development toward safer, more predictable nanomedicine platforms.^113–116^

## 6 Challenges, strategies, and future prospects for InP quantum dots

InP QDs represent a promising class of non-toxic nanomaterials with tunable optical properties, positioning them as alternatives to heavy-metal-based QDs for biomedical applications. Despite significant progress in their synthesis, surface engineering, and biological performance, several challenges remain that limit their clinical translation and widespread adoption. This section critically examines the current limitations in synthesis, stability, and biomedical applications of InP QDs, proposes innovative strategies to enhance their performance and biocompatibility, and explores their potential in emerging biomedical fields.

### 6.1 Current limitations in synthesis and applications

#### 6.1.1 Synthesis challenges

Current methods for synthesizing InP QDs face critical trade-offs between high photoluminescence quantum yield (PLQY), narrow emission linewidths, and monodispersity. For instance, hot-injection approaches can achieve PLQYs up to 97.7%, but their reliance on toxic HF for InPO_*x*_ defect removal raises safety concerns. In contrast, one-pot methods are safer and scalable but generally produce lower PLQYs and broader size distributions. Doping strategies, such as Nd^3+^ incorporation to achieve blue emission, enhance core stability but reach only 44% PLQY, illustrating a compromise between color tunability and optical efficiency.^15,37^ This critical comparison indicates that no single method simultaneously optimizes all key parameters, and method selection must be application-specific.

#### 6.1.2 Surface and stability issues

Surface defects, including dangling bonds and oxidized phosphorus species, significantly reduce PLQY and stability. Core/shell structures like InP/ZnSe/ZnS improve PLQY and mitigate lattice mismatch, yet shelling may produce secondary ZnS nanoparticles that compromise sample homogeneity. Surface modifications, such as PEGylation or ligand exchange with sulfide ions, enhance water dispersibility but often fail to maintain long-term stability under physiological conditions. Comparative analysis shows that while each surface strategy addresses specific limitations, a combination of approaches (*e.g.*, gradient-alloyed shells plus hybrid organic–inorganic ligands) is required to achieve simultaneous PLQY enhancement, stability, and reduced toxicity.^24,57^

#### 6.1.3 Biomedical application limitations

In biomedical applications, InP QDs face challenges related to targeting specificity, clearance, and physiological stability. Persistent accumulation in liver and spleen, dose-dependent toxicity, and non-specific binding are common issues. Critical comparison across studies reveals that surface engineering, such as functionalization with aptamers or zwitterionic ligands, improves cellular specificity and reduces organ retention, but standardization is lacking. Therefore, design choices in synthesis and surface chemistry directly dictate biomedical performance, highlighting the need for generalized principles to guide future development.^71,95^

### 6.2 Strategies for improving performance and biocompatibility

#### 6.2.1 Optimizing synthesis techniques

Safer and scalable synthesis methods are essential to overcome current limitations. Replacing HF with bifunctional metal oleates can increase PLQY (∼50%) and reduce emission linewidths by 20%. Non-pyrophoric phosphorus precursors, such as solid-state acylphosphines, improve uniformity and emission tunability from 460–600 nm. Machine learning-assisted optimization enables precise prediction of emission wavelengths and reaction conditions. Comparative analysis shows that slowing precursor reactivity, controlled doping, and real-time monitoring consistently improve both optical performance and reproducibility. However, trade-offs remain: high PLQY often requires complex shell engineering, while simple scalable methods yield moderate performance.^82,102^

#### 6.2.2 Advanced surface engineering

Robust surface engineering enhances both stability and biocompatibility. Silica encapsulation, hybrid organic–inorganic passivation, and gradient-alloyed shells improve colloidal stability and reduce toxicity, yet none alone provides a universal solution. Critical synthesis-surface comparisons indicate that integrating multiple strategies—ligand optimization, alloyed shells, and aptamer conjugation—achieves the most balanced performance, improving *in vivo* stability, PLQY, and targeting specificity simultaneously.

#### 6.2.3 Enhancing biomedical performance

Targeting specificity and functional performance in biomedical applications depend strongly on surface engineering and QD architecture. CPP or aptamer conjugation, zwitterionic ligands, and photosensitizer integration enhance cellular uptake, reduce non-specific binding, and improve ROS generation for PDT. Comparative evaluation suggests that a combined design strategy, matching core/shell architecture to surface ligands and application-specific functionalization, consistently yields superior performance.^110,118^

### 6.3 Potential of InP QDs in emerging applications

InP QDs exhibit unique optical and biocompatibility properties, positioning them as transformative tools in emerging biomedical fields. Their tunable NIR emission, high PLQY, and non-toxic composition enable innovative applications in theranostics, neural interfaces, point-of-care diagnostics, and immunotherapy.

#### 6.3.1 Theranostic platforms

InP QDs hold immense potential in theranostics, integrating diagnostics and therapy for precision medicine. Biosensors like UCNP-p@InP-cRGD detect matrix metalloproteinase-2 (MMP2) in cancer cells, enabling early diagnosis and targeted drug delivery with high specificity.^35^ Conjugating InP/ZnS QDs with stimuli-responsive polymers, such as pH- or redox-sensitive hydrogels, could facilitate controlled drug release in tumor microenvironments, enhancing therapeutic efficacy while minimizing off-target effects. Their NIR emission in the 650–900 nm window supports deep-tissue imaging, critical for real-time monitoring of therapeutic outcomes.^38^ Incorporating InP QDs into multifunctional nanocomposites with magnetic or plasmonic nanoparticles could further enable multimodal imaging and hyperthermia-based therapies.^123^

#### 6.3.2 Neural interfaces

In neural interfaces, type-II InP/ZnO core/shell QDs induce hyperpolarizing bioelectrical currents at low power densities (4 µW cm^−2^), offering high-resolution photostimulation for retinal prostheses.^33^ Their type-II band alignment minimizes reabsorption losses, enhancing photostimulation efficiency. Integrating InP QDs with optogenetic tools, such as channelrhodopsins, could enable precise spatiotemporal modulation of neural activity, advancing neurotherapeutics for conditions like Parkinson's or epilepsy.^124^ Surface functionalization with neurotrophic factors could improve QD-neuron interactions, promoting long-term stability in neural environments.^125^

#### 6.3.3 Point-of-care diagnostics

Biotemplated InP/ZnSe/ZnS-aptamer quantum dots (QDAPTs) exhibit rapid binding kinetics (<5 min) and three-month stability in aqueous media, enabling point-of-care bacterial detection using handheld imaging devices with a detection limit of ∼10^3^ CFU mL^−1^.^55^ Expanding this platform to detect viral antigens (*e.g.*, SARS-CoV-2 spike proteins) or protein biomarkers (*e.g.*, cardiac troponins) could revolutionize rapid diagnostics for infectious and chronic diseases.^126^ Integrating InP QD-based biosensors with microfluidic chips could enhance sensitivity and portability, facilitating deployment in resource-limited settings.^127^

#### 6.3.4 Immunotherapy monitoring

InP QDs show promise in monitoring immunotherapy responses by targeting immune cell biomarkers. Functionalizing InP/ZnS QDs with antibodies against programmed death-ligand 1 (PD-L1) could enable real-time imaging of immune checkpoint expression in tumors, guiding personalized immunotherapy strategies.^128^ Their high PLQY and photostability support longitudinal tracking of immune cell dynamics, critical for assessing treatment efficacy.^4,30^

### 6.4 Economic aspects and market potential of InP QDs in biomedical applications

The translation of InP QDs from research to clinical and commercial biomedical applications hinges not only on technical advancements but also on their economic viability and market competitiveness. Unlike prior subsections that addressed synthesis challenges, surface engineering, biocompatibility, or emerging applications, this subsection focuses on the economic landscape of InP QDs, evaluating cost-benefit dynamics, market growth projections, and competitive positioning in the biomedical nanotechnology sector.

The economic appeal of InP QDs lies in their non-toxic composition, which reduces downstream costs associated with waste management and regulatory compliance compared to cadmium-based QDs. While synthesis costs for InP QDs remain moderate due to the use of indium and phosphorus precursors, advancements in continuous-flow reactors, as noted in recent studies, have lowered production expenses by optimizing yield efficiency and reducing precursor waste. These cost savings are critical for applications like point-of-care diagnostics, where affordability drives adoption in resource-limited settings. However, high initial investment in specialized equipment (*e.g.*, microreactors) and quality control systems poses a barrier to small-scale manufacturers, necessitating strategic partnerships to amortize costs. Unlike earlier discussions on synthesis scalability, this section emphasizes the economic trade-offs, such as balancing precursor quality with cost to maintain NIR emission (650–900 nm) suitable for clinical imaging.^31,54^

The global market for quantum dots in biomedical applications is projected to grow significantly, driven by demand for non-toxic nanomaterials in diagnostics and theranostics. InP QDs are well-positioned to capture this market due to their cadmium-free composition and compatibility with NIR-based imaging, which is increasingly adopted in precision medicine. Their potential in high-sensitivity diagnostic kits (*e.g.*, detecting biomarkers at <5 pM) and theranostic platforms (*e.g.*, combining PDT with imaging) aligns with market trends favoring multifunctional nanomaterials. Unlike the application-focused discussion in 6.3, this analysis highlights market drivers, such as the rising prevalence of chronic diseases and the shift toward personalized medicine, which amplify demand for InP QD-based products.^17,48^

InP QDs face competition from established nanomaterials like gold nanoparticles (used in plasmonic diagnostics) and carbon dots (favored for low-cost sensors). However, their unique combination of non-toxicity, tunable NIR emission, and photostability provides a competitive edge in applications requiring deep-tissue imaging or long-term stability, such as intraoperative fluorescence guidance. To strengthen market positioning, manufacturers must address economic challenges, including optimizing supply chains for high-purity precursors and reducing costs through localized production. Collaborative models, such as public-private partnerships or licensing agreements with diagnostic companies, can accelerate market entry. This contrasts with prior subsections' focus on technical comparisons, as it evaluates InP QDs' economic viability against market competitors without revisiting performance metrics like LOD or PLQY.^39,60^

Key economic barriers include the high cost of preclinical and clinical trials required for regulatory approval, which can exceed millions of dollars per candidate. InP QDs' non-toxic profile may streamline safety assessments compared to cadmium-based alternatives, potentially reducing trial costs. However, variability in large-scale functionalization (*e.g.*, PEGylation or aptamer conjugation) increases production expenses, requiring investment in automated quality control systems. Opportunities lie in targeting high-value markets, such as oncology diagnostics, where InP QD-based kits could command premium pricing due to their sensitivity and safety.^72–74^ Additionally, integrating InP QDs into existing diagnostic platforms, like microfluidic devices, could lower adoption costs by leveraging established infrastructure.

### 6.5 Critical comparison and design principles

Despite extensive research on InP QDs, the literature often emphasizes individual studies without systematically comparing outcomes across synthesis methods, surface modifications, or applications. A critical synthesis of reported data highlights key trends and allows the formulation of generalized design principles for improved optical performance, stability, and biocompatibility.

#### 6.5.1 PLQY and optical performance

High PLQYs are consistently achieved with multi-shell architectures, particularly InP/ZnSe/ZnS structures, combined with optimized ligand passivation. Core-only or one-pot synthesized QDs tend to exhibit lower PLQYs and broader emission spectra, but these methods are more scalable and less hazardous. Doping strategies, including rare-earth ions or metal cations, can enhance emission color or stability but often introduce trade-offs in quantum efficiency. Comparative analysis indicates that high PLQY and narrow emission linewidths require carefully coordinated synthesis and shell engineering, whereas simple, scalable methods sacrifice peak optical performance.^101,114^

#### 6.5.2 Stability trends

QDs with gradient-alloyed shells and hybrid organic–inorganic ligands show superior stability in aqueous and physiological conditions. Single-shell systems or unmodified surfaces often degrade rapidly, leading to loss of emission intensity and potential toxicity. Surface engineering strategies, such as PEGylation, improve solubility but do not fully prevent long-term degradation or organ accumulation *in vivo*. Cross-study comparison demonstrates that combining alloyed shells with functional ligands achieves the most consistent enhancement in both colloidal and biological stability**.**

#### 6.5.3 Toxicity and biocompatibility

Surface functionalization strongly dictates biological responses. Zwitterionic ligands and aptamer conjugation reduce non-specific uptake by liver and spleen, mitigating toxicity, while unpassivated QDs or those with simple carboxylation display higher organ retention and inflammatory responses. Core/shell structures limit In^3+^ ion leaching, underscoring the interdependence of core design, shell architecture, and ligand chemistry in determining safety profiles.^95,103^

#### 6.5.4 Design principles

From these comparative insights, several generalized guidelines emerge:

(1) Core/shell engineering: employ multi-shell or gradient-alloyed structures to optimize PLQY, emission linewidth, and lattice mismatch.

(2) Ligand selection: choose ligands based on intended application—PEGylation for stability, zwitterionic or aptamer-based for targeted delivery, and CPPs for enhanced cellular uptake.

(3) Synthesis control: slow, controlled nucleation and growth improve monodispersity and optical properties while maintaining scalability.

(4) Integrated approach: optimal performance requires the combined design of core/shell architecture, surface passivation, and functionalization rather than isolated modifications.

(5) Application-specific tuning: adjust emission wavelengths, surface chemistry, and functional groups to meet biomedical objectives, such as deep-tissue imaging, photodynamic therapy, or rapid diagnostics.

This comparative framework shifts focus from isolated experimental reports to principled, predictive design**,** enabling the rational development of next-generation InP QDs that maximize performance, stability, and biocompatibility in diverse biomedical applications (Table 12).

**Table 12:** Economic aspects and market potential of InP QDs in biomedical applications

Aspect	Current status	Economic challenges	Opportunities	Market potential	Ref.
Cost-benefit dynamics	Moderate synthesis costs; reduced waste management	High equipment investment	Continuous-flow optimization; partnerships	High (cost-sensitive diagnostics)	31 and 54
Market growth	Projected $8–10B by 2030	Competition from established nanomaterials	NIR-based diagnostics; theranostics	High (oncology, personalized medicine)	17 and 48
Competitive positioning	Strong due to non-toxicity, NIR emission	Supply chain costs	Localized production; licensing	High (deep-tissue imaging)	39 and 60
Regulatory costs	High for clinical trials	Trial funding barriers	Streamlined safety assessments	Moderate (pending approvals)	34 and 72
Commercial adoption	Emerging in diagnostic kits, theranostics	Functionalization costs	Integration with existing platforms	High (point-of-care devices)	59 and 78

## 7 Conclusion

InP QDs represent a transformative platform in biomedical nanotechnology due to their non-toxic nature, tunable near-infrared emission, and high PLQYs up to 97.7%. This review highlights advanced synthesis strategies, including hot-injection and one-pot methods, alongside surface engineering techniques like ligand exchange and PEGylation, enhancing stability and biocompatibility. InP QDs excel in multiplexed bioimaging (515–845 nm), biosensing (∼10^3^ CFU mL^−1^ detection), and photodynamic therapy (>99.9% bacterial inactivation), with applications in drug delivery and sneural prosthetics. Low cytotoxicity is confirmed, though indium release and organ accumulation pose challenges. Future efforts should focus on safer precursors and zwitterionic ligands to improve monodispersity, stability, and clearance. This pioneering review, focused on InP QDs in biomedical contexts, lays a foundation for their clinical translation, positioning them as a cornerstone for precision nanomedicine in diagnostics and therapeutics.

## Author contributions

A. K. Kareem and Nadia Sarhan conceived the scope and structure of the review. A. K. Kareem, Musallam Ahmed Salim Tabook, and Esraa H. J. Mahdi performed the literature survey and data collection. Ahmed Said Badawy, Rekha M. M, and Laxmidhar Maharana contributed to critical analysis of synthesis strategies and surface engineering concepts. P. Grace Kanmani Prince, Gaganjot Kaur, and Hamza Fadhel Hamzah contributed to the sections related to biomedical imaging, sensing, and therapeutic applications. Nadia Sarhan coordinated the manuscript preparation and correspondence. All authors contributed to manuscript revision, participated in scientific discussion, and approved the final version for publication.

## Conflicts of interest

There are no conflicts to declare.

## Data Availability

No primary research results, software or code have been included and no new data were generated or analysed as part of this review.
